# Progression of monoclonal gammopathy of undetermined significance to multiple myeloma is associated with enhanced translational quality control and overall loss of surface antigens

**DOI:** 10.1186/s12967-024-05345-x

**Published:** 2024-06-07

**Authors:** Sigrid Ravn Berg, Aida Dikic, Animesh Sharma, Lars Hagen, Cathrine Broberg Vågbø, Alexey Zatula, Kristine Misund, Anders Waage, Geir Slupphaug

**Affiliations:** 1 Department of Clinical and Molecular Medicine, Norwegian University of Science and Technology, NTNU, N-7491 Trondheim, Norway; 2 Clinic of Laboratory Medicine, St. Olavs hospital, N-7491 Trondheim, Norway; 3 PROMEC Core Facility for Proteomics and Modomics, Norwegian University of Science and Technology, NTNU, and the Central Norway Regional Health Authority Norway, N-7491 Trondheim, Norway; 4 Department of Medical Genetics, St Olavs hospital, N-7491 Trondheim, Norway; 5 Department of Hematology, and Biobank1, St Olavs hospital, N-7491 Trondheim, Norway

**Keywords:** Mutiple myeloma, MGUS, Proteomics, Translational quality control, EIF2, Integrin, MYC, TGFB1

## Abstract

**Background:**

Despite significant advancements in treatment strategies, multiple myeloma remains incurable. Additionally, there is a distinct lack of reliable biomarkers that can guide initial treatment decisions and help determine suitable replacement or adjuvant therapies when relapse ensues due to acquired drug resistance.

**Methods:**

To define specific proteins and pathways involved in the progression of monoclonal gammopathy of undetermined significance (MGUS) to multiple myeloma (MM), we have applied super-SILAC quantitative proteomic analysis to CD138 + plasma cells from 9 individuals with MGUS and 37 with MM.

**Results:**

Unsupervised hierarchical clustering defined three groups: MGUS, MM, and MM with an MGUS-like proteome profile (ML) that may represent a group that has recently transformed to MM. Statistical analysis identified 866 differentially expressed proteins between MM and MGUS, and 189 between MM and ML, 177 of which were common between MGUS and ML. Progression from MGUS to MM is accompanied by upregulated EIF2 signaling, DNA repair, and proteins involved in translational quality control, whereas integrin- and actin cytoskeletal signaling and cell surface markers are downregulated.

**Conclusion:**

Compared to the premalignant plasma cells in MGUS, malignant MM cells apparently have mobilized several pathways that collectively contribute to ensure translational fidelity and to avoid proteotoxic stress, especially in the ER. The overall reduced expression of immunoglobulins and surface antigens contribute to this and may additionally mediate evasion from recognition by the immune apparatus. Our analyses identified a range of novel biomarkers with potential prognostic and therapeutic value, which will undergo further evaluation to determine their clinical significance.

**Supplementary Information:**

The online version contains supplementary material available at 10.1186/s12967-024-05345-x.

## Background

Multiple myeloma (MM) is characterized by uncontrolled growth of monoclonal plasma cells (myeloma cells) in the bone marrow and production of a monoclonal immunoglobulin (the M-component). MM is the second most common hematological malignancy in high-income countries and in 2020 it was estimated 176 404 new cases worldwide [1]. The median age at diagnosis of MM is about 70 years. The global incidence rate and deaths by MM have more than doubled in the last 30 years and with population aging, the incidence rate is projected to continue rising [2]. Novel therapeutic agents such as immunomodulatory drugs, proteasome inhibitors (PIs) and antibodies targeting cell surface antigens, as well as autologous stem-cell transplantation (ASCT), have markedly improved overall survival (OS), which is currently about 10 years for patients eligible for ASCT [3]. Nevertheless, most patients develop drug resistance and eventually die from the disease. The etiology of MM is poorly understood, but almost all cases are preceded by an asymptomatic pre-malignant disorder called Monoclonal Gammopathy of Undetermined Significance (MGUS) [4]. MGUS occurs in ~ 3% of the population over the age of 50 and 5% over the age of 70, and incidence increases with advancing age. The risk of progression from MGUS to MM is ~ 1% per year [5]. Although MGUS and MM apparently share many of the primary genetic abnormalities involved in disease initiation, such as *IgH* translocations, aneuploidy, chromosome 13 deletion, and dysregulation of cyclin D, secondary or cooperating events are probably required.

We can describe clinical criteria defining the transition from MGUS to myeloma, however, precise understanding of the underlying biological mechanisms leading to this transition is still lacking. It is thus difficult to predict if and when MGUS will progress to MM in individual patients. MGUS patients can generally be risk stratified based on abnormal serum free light chain (kappa/lambda) ratio (sFLC), monoclonal protein (MCP) concentration and isotype and bone marrow plasma cell quantification [6]. However, the accuracy of this stratification is limited by a number of factors [7]. In addition, DNA aneuploidy and immunoparesis (reduced normal serum immunoglobulins) were found to be independent predictors of progression to MM [8]. Some studies have attempted to identify mRNA expression signatures that might predict progression of MGUS to MM [6, 9–12]. These have reported enrichment in MYC-, E2F- and chromosomal instability pathways [11] and in anti-apoptotic, NF-kB, DNA repair, and cytokines signaling pathways [12]. Whereas such studies may have predictive value, their potential to identify druggable targets is limited since there is generally a poor overall correlation between mRNA expression and the corresponding protein products [13]. Few studies have attempted to identify MM biomarkers based on proteomic data. Chankuppa et al. [14] analyzed patient-derived MM mononuclear cells (MNCs) and identified marginal zone B and B1 cell specific protein (MZB1) as a potential driver of MM pathogenesis. In a study of plasma cells from newly diagnosed MM patients and healthy controls, Wu et al. [15] found oxidative metabolism and protein synthesis to be most upregulated in the malignant cells and identified 60 kDa mitochondrial heat shock protein HSPD1 as a potential therapeutic target for MM treatment. In a surfaceome study of captured N-linked glycoproteins from four MM cell lines, Ferguson et al. [16] identified 530 proteins that were localized to the surface with high confidence and identified several proteins that could serve as markers for treatment responses. Our group correlated activity levels of selected MM signaling proteins in nine MM cell lines, with response to 33 targeted drugs, showing that signaling protein profiling holds promise to predict drug sensitivity in MM [17]. Finally, Yao et al. [18] recently employed a multi-omic pipeline to identify novel MM therapeutic targets, which included single-cell RNA-seq of bone marrow aspirates from 41 MM patients. This revealed 38 MM marker genes encoding surface proteins, and 15 encoding intracellular proteins, which were cross validated by flow cytometry and MS bulk proteome profiling in four MM cell lines and four MM patient samples.

To the best of our knowledge, there are no published reports of studies that might predict progression of MGUS to MM at the whole-proteome level. Here, we present a super-SILAC-based quantitative proteome analysis of purified CD138 + plasma cells obtained from MGUS and MM patients. Stable isotope labeling with amino acids in cell culture (SILAC) is a simple, robust, and accurate quantitative proteomics method that has been widely applied to characterize protein changes between different samples [19]. The development of super-SILAC as a common internal standard allows the comparison of samples that cannot be metabolically labeled, such as clinical tumor samples [20]. By using a labeled reference library generated from cell lines, super-SILAC enables accurate and reproducible quantification of protein expression levels across different experimental conditions. We have previously performed super-SILAC quantitative proteome profiling of malignant plasma cells collected from the same patient at both the MM and secondary plasma cell leukemia (sPCL) stages of the disease to elucidate factors contributing to transformation [21]. Here we have analyzed CD138 + cells isolated from nine MGUS and 37 MM clinical patient samples and achieved a quantitative depth of more than 6000 proteins. We show the segregation of MGUS and MM clinical samples based on their total protein expression profiles and point to key canonical pathways apparently associated with early and late steps in the progression of MGUS to MM. Our results are novel and may be of value for identification of new treatment targets, prognostic characterization, and stratification of patients for differential treatment.

## Methods

### Patient material and cell culture

This study analyzed 37 patients with MM and nine with MGUS from Norwegian hospitals between 2001 and 2015 (Supplementary Table 1). Clinical data for each patient were collected at the time of diagnosis. Diagnostic criteria were, with some modifications, based on the 2003 guidelines [22]. In short, diagnosis of MGUS was given when monoclonal immunoglobulin was < 30 g/l and bone marrow clonal plasma cells were < 10% with no evidence of multiple myeloma. In smoldering myeloma, the M protein was ≥ 30 g/l and/or bone marrow clonal cells ≥ 10% but not increased calcium, renal insufficiency, anemia, or bone lesions (CRAB criteria) attributed to the plasma cell proliferative process. Symptomatic myeloma requiring treatment met one or more CRAB criteria. Median age at diagnosis was 63.5 with 54% male cases. Malignant CD138 + cells were isolated and stored as described [23].

Carfilzomib-sensitive (AMO1) and resistant (AMO1-CFZ) MM cells were grown in RPMI-1640 medium (Sigma-Aldrich) supplemented with 10% FBS (Sigma-Aldrich) and 0.6 mM L-glutamine, in a humidified incubator at 5% CO2 and 37 °C. The AMO1-CFZ medium was additionally added 90 nM CFZ, while the CFZ sensitive AMO1 cells were added vehicle (0.009% DMSO) only. One week prior to harvest, AMO1-CFZ cells were grown in the absence of CFZ (vehicle only).

### Sample preparation and proteomic analysis

We previously described the heavy super-SILAC library for quantitative proteome profiling [21]. For super-SILAC analyses, cells were washed three times in PBS, lysed in 7 M urea, 2 M thiourea, 2.5% CHAPS, 25 mM DTT, and homogenized using Kontes™ Pellet Pestle™ Grinder. After thorough vortexing, homogenates were incubated for 15 min at room temperature (RT) and centrifuged at 16,000 × *g* for 15 min at RT. Protein concentrations in supernatants were determined by the Bradford method (Bio-Rad Laboratories Inc, Hercules, CA). 25 μg of protein from each patient sample was mixed 1:1 with the super-SILAC library. Proteins were precipitated by methanol/chloroform, trypsin digested, and dissolved peptides analyzed on an Easy-nLC 1000 UHPLC system (Thermo Scientific/Proxeon) interfaced with an LTQ-Orbitrap Elite hybrid mass spectrometer (Thermo Scientific) via a nanospray ESI ion source (Proxeon, Odense). All samples were analyzed in at least three technical replicates, and some samples were rerun in different batches to monitor potential batch effects (Supplementary Fig. 1). Peptides were injected onto a C-18 trap column (Acclaim PepMap100, 75 μm i. d. × 2 cm, C18, 5 μm, 100 Å, Thermo Scientific) and further separated on a C-18 analytical column (Acclaim PepMap100, 75 μm i. d. × 50 cm, C18, 3 μm, 100 Å, Thermo Scientific). The LC was operated at 250 nL/min over 262 min with solvent A consisting of 0.1% formic acid (FA) in water and solvent B of 0.1% FA in CH_3_CN. Peptides were eluted with a linear gradient of 0–30% solvent B over 252 min, followed by 5 min at 100% B and 5 min at 100% A. Peptides were analyzed in positive ion- and data dependent acquisition (DDA) mode using electrospray voltage 2.2 kV, CID fragmentation with normalized collision energy 35, automatic gain control target value of 1E6 for Orbitrap MS and 1E4 for MS/MS scans. Each MS scan (m/z 400–1600) was acquired at 120,000 FWHM, followed by 20 MS/MS scans triggered for intensities above 500 and selected with an isolation window of 2 Th, at a maximum ion injection time of 200 ms for MS and 50 ms for MS/MS scans.

AMO1 and AMO1-CFZ cells were resuspended in 100 µl 1% sodium deoxycholate, 100 mM Tris–HCl pH 8.5, 10 mM tris(2-carboxyethyl)phosphine (TCEP), 40 mM chloroacetamide (CAA), heated at 90 °C for 45 min and sonicated for 10 cycles (30 s ON/30 s OFF) using a Bioruptor pico sonicator. After centrifugation at 16,000 × *g* for 10 min, 50 µg soluble protein from each sample was added 100 µl 0.1 M ammonium bicarbonate, 0.5 µg trypsin and digested overnight at 37 °C. Peptides were desalted using C18 spin columns, dried in a speedvac and resuspended in 0.1% FA prior to MS analysis. Label-free quantitative (LFQ) LC–MS/MS was performed on a timsTOF Pro (Bruker Daltonics) connected to a nanoElute (Bruker Daltonics) HPLC. Peptides were separated over a Bruker PepSep C18 (75 µm × 15 cm) column with running buffers A (0.1% formic acid) and B (0.1% FA in acetonitrile) using a 100 min gradient from 2% B to 40% B. The timsTof was operated in the DDA PASEF mode with 10 PASEF scans per acquisition cycle and accumulation and ramp times of 100 ms each. The ‘target value’ was set to 20000 and dynamic exclusion was activated and set to 0.4 min. The quadrupole isolation width was set to 2 Th for m/z < 700 and 3 Th for m/z > 800.

### Bioinformatic analyses

Super-SILAC raw files were analyzed with MaxQuant v 1.6.0.1 [24] using its default settings with multiplicity 2 (Arg10, Lys8), carbamidomethyl as fixed modification of cysteine, methionine oxidation/protein N-terminal acetylation/asparagine and glutamine deamidation as dynamic modifications, PSM/sitep FDR of 0.01, FTMS and ITMS MS/MS tolerance of 0.5 Da and 20 ppm, respectively. Search was performed against the April 2017 version of the Human proteome set with isoforms from UniProt [http://www.uniprot.org/proteomes/UP000005640], and gene/protein names manually curated against the January 2023 version of Human proteome set with isoforms from UniProt. The values from super-SILAC ratios were analyzed by Perseus software v 1.6.15 [25]. Values from technical replicates were transformed to log_2_ to give a better approximation to normal distribution of super-SILAC ratios. The median of technical replicates was calculated to represent the samples, for reduced sensitivity to outliers. Protein groups were filtered based on a criterion requiring at least 60% values in one of the groups followed by imputing the missing values to reflect the detection limit of the instrument, namely downshifting the overall mean of the resultant matrix by 0.5 and scaling the standard deviation by 0.3 [26] (Supplementary Figure 2). Ingenuity^®^ Pathway Analysis (IPA) (Ingenuity Systems, www.ingenuity.com) and PANTHER overrepresentation test (Release 20221013, GO Ontology database 10.5281/zenodo.6799722 Released 2022-07-01) was used to search for modified biological pathways in the datasets of proteins selected by statistical analysis. The CoMMpass data (https://gdc.cancer.gov/about-gdc/contributed-genomic-data-cancer-research/foundation-medicine/multiple-myeloma-research-foundation-mmrf) were collected and processed for the 995 cases with clinical observations using MMRF-CoMMpass package (MMRF-CoMMpass Data Integration and Analysis for Identifying Prognostic Markers). The Survival-analysis thresholds used for High and Low expression were 0.76 and 0.33, respectively, following the package guidelines (https://github.com/marziasettino/MMRFBiolinks/blob/master/vignettes/Analysis.Rmd), and the univariate Kaplan–Meier plots were modified to display complete curves. The code for the modified plot and app deployed with other details are available at the package fork (https://github.com/animesh/MMRFBiolinks).

Label-free MS data were analyzed using MaxQuant v.2.3.1.0 [27]. Open workflow [28] was used to inspect the raw files to determine optimal search criteria, namely: enzyme specified as trypsin with a maximum of two missed cleavages allowed; acetylation of protein N-terminal, oxidation of methionine, and deamidation of asparagine/glutamine as dynamic post-translational modification. These were imported in MaxQuant which uses m/z and retention time (RT) values to align each run against each other sample with a minute window match-between-run function and 20 min overall sliding window using a clustering-based technique. These were further queried against the Human proteome including isoforms downloaded from UniProt (https://www.uniprot.org) in February 2023 and MaxQuant’s internal contaminants database using Andromeda built into MaxQuant. Both Protein and peptide identifications false discovery rate (FDR) was set to 1%, and only unique peptides with high confidence were used for identification of final protein groups. Peak abundances were extracted by integrating the area under the peak curve. Each protein group abundance was normalized by the total abundance of all identified peptides for each run and protein by calculated median summing all unique peptide-ion abundances for each protein using label-free quantification (LFQ) algorithm [29] with minimum peptides ≥ 1. Normalized LFQ values of protein-groups for all samples were log_2_-transformed and compared using T-test between groups in R (https://cran.r-project.org/). The calculated p-values were corrected by the Benjamini Hochberg procedure, using R function p.adjust with “BH” as the method. Differentially expressed (DE) protein groups were identified at corrected-p-value < 0.05. Those protein-groups quantified exclusively in one group with coefficient-of-variation < 0.05 were assigned 0 as p-values.

### Quantification of RNA modifications by LC–MS/MS

Total RNA was isolated using mirVana™ miRNA Isolation Kit (Thermo Fisher), enzymatically digested using benzonase (Santa Cruz Biotech) and nuclease P1 (Sigma) in 10 mM ammonium acetate pH 6.0 and 1 mM MgCl_2_ at 40 °C for 1 h, added ammonium bicarbonate to 50 mM, phosphodiesterase I and alkaline phosphatase (Sigma) and incubated further at 37 °C for 1 h. Digested samples were precipitated with 3 volumes of acetonitrile and supernatants lyophilized and dissolved in Optima^™^ LC/MS grade water (Thermo Fisher). An Agilent 1290 Infinity II UHPLC system with a ZORBAX RRHD Eclipse Plus C18 150 × 2.1 mm (1.8 μm) column protected with a ZORBAX RRHD Eclipse Plus C18 5 × 2.1 mm (1.8 µm) guard (Agilent) was used for chromatographic separation. The mobile phase consisted of A: water and B: methanol (both added 0.1% FA) at 0.22 ml/min, for modifications starting with 5% B for 0.5 min followed by 2.5 min of 5–20% B, 3.5 min of 20–95% B, and 4 min re-equilibration with 5% B. Canonical nucleosides were chromatographed with a 4 min gradient of 5–95% B and 4 min re-equilibration with 5% B. Mass spectrometric detection was performed using an Agilent 6495 triple quadrupole system monitoring the mass transitions 268.1–136.1 (A), 284.1–152.1 (G), 244.1–112.1 (C), 245.1–113.1 (U), 296.1–150.1 (m^6^Am) and 326.1–194.1 (m^2,2,7^G) in positive ionization mode.

## Results and discussion

### Unsupervised hierarchical clustering identifies three disease groups

**Fig. 1:**
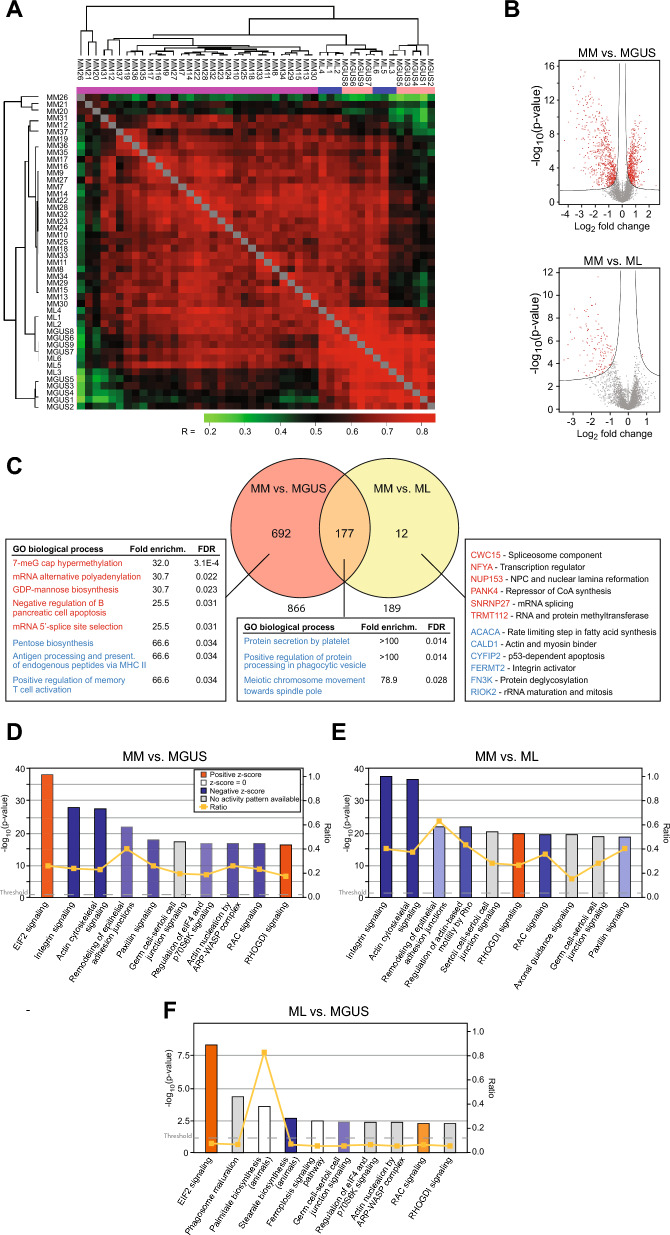
**A** Hierarchical clustering of the proteome data identifies a subgroup of MM that we denote ML (blue) that segregates with MGUS (peach). **B** Volcano plots of DEPs in MM vs. MGUS (upper panel) and MM vs. ML (lower panel). **C** Venn diagrams showing the number of distinct and overlapping DEPs in the two groups in (B). Enriched upregulated (red) and downregulated (blue) biological processes as reported by PANTHER GO analysis are boxed. In the MM vs. ML group, all distinct DEPs and their known functions are given. **D** Most affected biological pathways in MM vs. MGUS as reported by IPA (q < 0.05, log_2_ difference >|0.58|). **E** Most affected biological pathways in MM vs. ML as reported by IPA (q < 0.05, log_2_ difference >|0.58|). **F** Most affected biological pathways in ML vs. MGUS as reported by IPA (p < 0.05, log_2_ difference >|0.58|)

Combined analysis of the raw MS data by MaxQuant resulted in super-SILAC ratios for 6067 protein groups across the 46 patient samples, with an average of 3941 per sample. To compare the MGUS and MM proteomes, all measurements were quantified against each other based on the ratios to the super-SILAC library and their correlation coefficients calculated. To investigate whether our data could segregate MGUS from MM proteomes and to determine an optimal data analysis strategy, we performed unsupervised hierarchical clustering of all proteome measurements. We required that proteins were present in at least 60% of the measurements and filled any missing values by data imputation [25]. The clustering algorithm grouped together all the replicates of each sample, implying high technical reproducibility in our LC–MS/MS analysis. The hierarchical correlation matrix distinguished MGUS from MM, but also showed co-clustering of MGUS and six MM samples (Fig. 1A, blue in top row). One of these patients (MM6) fulfilled the criteria for smoldering myeloma. These six MM samples were designated as MGUS-like MM (ML). Although a significant proportion of patients in our cohort, especially in MGUS and ML, are still alive, Kaplan-Meyer analysis indicates that ML may have a favorable prognosis compared to MM (Supplementary Fig. 3). It should be noted that a group of MGUS-like MM was described in a previous study based on transcriptome profiling [9]. However, their differentially expressed gene (DEG) profile shares little overlap with the DEPs observed by protein profiling, underscoring the poor mRNA/protein correlation previously observed in MM cells [16]. Overall, 5215 protein groups were quantified in MGUS, 5207 in ML and 5895 in MM. From these, 2029 were quantified in all 46 samples, and on the average 5 proteins were exclusive to each sample (Supplementary Table 2) and separately compared with the MM samples using Student’s T-test followed by permutation based false discovery rate (FDR) correction (marked red in volcano plots, Fig. 1B, the curves represent the S0 threshold 0.1 [30]).

To identify proteins and pathways involved in progression from MGUS to MM, we looked for differentially expressed proteins (DEPs, q-value < 0.05, log_2_ difference >|0.58|) in each of the three categories MM vs. MGUS, MM vs. ML and ML vs. MGUS. A total of 866 DEPs were identified between MM vs. MGUS, with 400 proteins upregulated and 466 downregulated. 189 DEPs were identified in MM vs. ML, with 9 upregulated and 180 downregulated (Fig. 1C). No proteins displayed significant differential expression in ML vs. MGUS by using the same statistical criteria. Among the DEPs, we found an overlap of 177 proteins between ML and MGUS when compared to MM and all of these were downregulated in MM compared to the two other groups. PANTHER Gene Ontology (GO) analysis of the overlapping DEPs reported no upregulated processes, and the following biological processes as most downregulated: Protein secretion by platelet, Positive regulation of protein processing and Phagocytic vesicles and meiotic chromosome movement towards spindle pole (Fig. 1C, middle box). Analysis of the DEPs that were unique to MM vs. MGUS reported 7-meG cap hypermethylation and mRNA alternative polyadenylation as the most upregulated biological processes, whereas the most downregulated processes were Pentose biosynthesis and Antigen processing and presentation of endogenous peptides via MHC II (Fig. 1C, left box). No specific biological process was reported for the 12 DEPs unique to MM vs. MGUS (Fig. 1C, right box). Strikingly, when the DEPs were mapped on GO molecular functions, RNA binding was most significantly enriched among the upregulated proteins in MM vs. MGUS (7.0 -fold, FDR = 6.6E–127) whereas cytoskeletal protein binding was most enriched among the downregulated proteins (7.6-fold, FDR = 1.2E–35). We also employed GOrilla analysis (https://cbl-gorilla.cs.technion.ac.il/) by employing ranked, unfiltered gene names as input. This largely recapitulated the results from PANTHER analysis (data not shown), suggesting that filtering did not mediate artificial skewing of the reported processes.

Finally, we employed Ingenuity^®^ Pathway Analysis (IPA) to search for modified biological pathways in each of the subgroups. Gene identifiers of the DEPs were mapped in IPA and plotted onto canonical pathways. The ten most significantly affected pathways are shown in Fig. 1D–F. Here, EIF2 signaling was reported most upregulated (z-score = 3.48, p = 5.93E-39) and Integrin signaling most downregulated (z-score = − 5.86, p = 1.34E-28) in MM vs. MGUS (Fig. 1D). The pattern of affected pathways in MM vs. ML was very similar. The most notable exception was that EIF2 signaling was missing (Fig. 1E). Moreover, whereas activation of MYC (not quantified in our dataset) was predicted to be the top upstream regulator in MM vs. MGUS (activation z-score = 5.64, p = 3.04E-39), downregulation of TGFB1 (2.1-fold downregulated, activation z-score − 4.58) was predicted the top upstream regulator in MM vs. ML. Based on this, we hypothesize that activation of MYC and EIF2 signaling might be common early events in the transformation of MGUS to MM, whereas downregulation of TGFB1 and integrin signaling are late events. To investigate this further, we re-analyzed the ML vs. MGUS data with relaxed statistical criteria (p-value < 0.05, log_2_ difference >|0.58|). In support of our hypothesis, this reported EIF2-signaling as top affected pathway (z-score 2.71) (Fig. 1F) and MYC as top upstream activator (z-score = 2.24, p = 4.96E-10), whereas Integrin signaling was not significantly affected. Noteworthy, a recent study from our group analyzing MM clonal evolution resulting from treatment pressure, also identified increased expression of MYC- and decreased expression of TGFB pathways in late, compared to early stages of MM [31].

*MYC* alterations are rare in MGUS but occur in over 40% of newly diagnosed MM [32]. A gene expression study identified a MYC activation signature in about 67% of MM cases but not in normal plasma cells or MGUS [33]. MYC overexpression is also an independent risk factor for progression from SMM to MM [32, 34]. Finally, induction of MYC deregulation in germinal center B cells of mice with MGUS-like features led to full-blown MM [35]. A very recent CRISPR/Cas9 screen identified the deubiquitinase OTUD6B as a central mediator of MYC expression in MM cell lines. However, we find no significant difference in OTUB6B across the patient groups in our study, indicating that this mechanism might not be relevant in the tumor setting. More likely, MYC is upregulated via the transcription factor IRF4 [36], which was two-fold upregulated in (p = 1.7E-7) in MM vs. MGUS and also modestly but significantly upregulated in MM vs. ML. IRF4 is indirectly targeted by IMiDs such as thalidomide and lenalidomide since they alter target specificity of the CUL4A-DDB1-Cereblon E3 ubiquitin ligase. This leads to degradation of the plasma cell transcription factors IKZF1 (Ikaros) and IKZF3 (Aiolos) and downregulation of IRF4 [37, 38]. Notably, both IKZF1 and IKZF3 were significantly upregulated in MM vs. MGUS as well as in MM vs. ML, suggesting that increased MYC/IRF4 activation is an ongoing process during MM progression. MYC is suppressed by TGFB1, which acts as a tumor suppressor across several cancers [39]. TGFB1 was very significantly downregulated in MM vs. MGUS (2.8-fold, p = 1.3E-5) as well as in MM vs. ML (2.1-fold, p = 0.001). Downregulated TGFB1 signaling in the MM cells was further supported by the lack of detection of any TGFβ receptors as well as a marked downregulation of TGFB1-induced protein TGFB1I1 (3.5-fold, p = 9.9E-5). Finally, the integrins that convert latent TGFβ into the bioactive form were either not detected (ITGAV, ITGB6) or strongly downregulated (ITGB3, ITGB5) in MM vs. MGUS.

### MM cells produce decreased levels of immunoglobulin compared to premalignant MGUS cells

Most MM cells produce large quantities of immunoglobulins, mediating high demand on their protein-handling machinery. This has also contributed to explaining the remarkable effectiveness of proteasome inhibitors in the treatment of MM [40]. Whereas this holds true for MM compared to most other cancers, it might not characterize MM cells compared to premalignant plasma cells in MGUS. Among the 14 quantified IGH, -K- and L chains, nine showed significant differential expression, all of which were downregulated (1.4–6.1-fold) in MM vs. MGUS. This strongly suggests that the M-spike increase in MM progression is due to an increasing number of MM cells rather than increased M-protein synthesis per cell. Immunoglobulins are large and complex proteins that require chaperones for correct folding and extensive post-translational modification in the ER. Reducing the synthesis of M-protein per cell would thus reduce the risk of protein aggregation and induction of the unfolded protein response (UPR). In support of the latter, the UPR markers EIF2AK3, ERN1 and ATF6 remained below the detection level in both MM and MGUS.

### Progression from MGUS to MM is accompanied by enhanced ribosomal biogenesis and epitranscriptomic writers, potentially augmenting translational fidelity

**Fig. 2:**
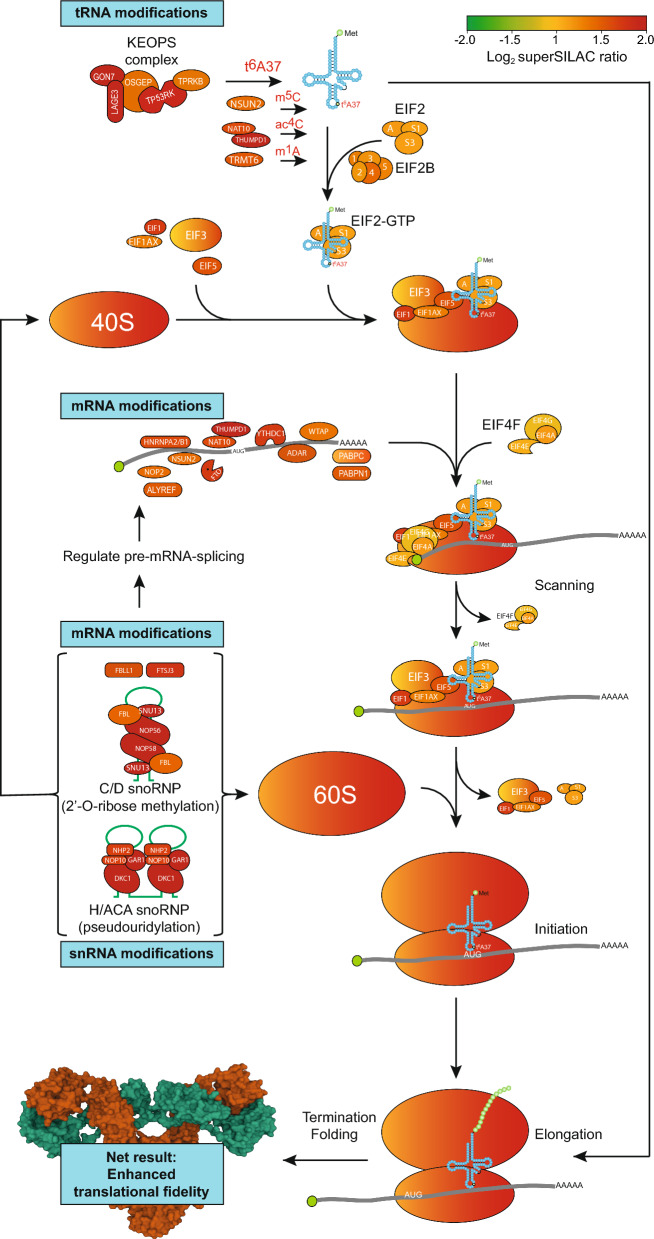
Proteins associated with EIF2 signaling and protein translation were markedly upregulated in MM vs. MGUS (scale bar at top). Among the most upregulated DEPs were proteins involved in modification of tRNAs, rRNAs mRNAs and snRNAs. Many of these modifications are known to induce RNA modifications that induce protein translational fidelity and that may be highly significant to minimize formation of aberrant proteins and induction of UPR in MM cells (shown is a human IgG1, PDB 1MCO). The RNA parts of the C/D and H/ACA snoRNPs are in green

EIF2 signaling is the master regulator of ribosomal biogenesis and protein synthesis. Of the 75 human cytoplasmic ribosomal proteins (RPs) [41], 71 were found to be significantly upregulated in MM vs. MGUS (1.2–3.3-fold, p-values from 3.7E-2 to 2.0E-9) (Supplementary Table 2, Fig. 2). We also found overall upregulation of mitochondrial ribosomal proteins (MRPs). Of the 64 MRPs quantified in our dataset, 26 were significantly differentially expressed (p < 0.05) and all were upregulated.

Enhanced ribosome biogenesis has also been observed in other cancers but has been attributed to the need for increased protein production to support increased proliferation. This is apparently not the case in MM, since the proliferation markers MKI67, PCNA and MCM1-7 were not altered in MM vs. MGUS. We hypothesize that the increased number of ribosomes may instead affect protein quality by enabling reduced translational speed. When there are more ribosomes available in the cell, the overall workload can be distributed among a larger number of ribosomes, thereby mediating increased translational fidelity.

**Fig. 3:**
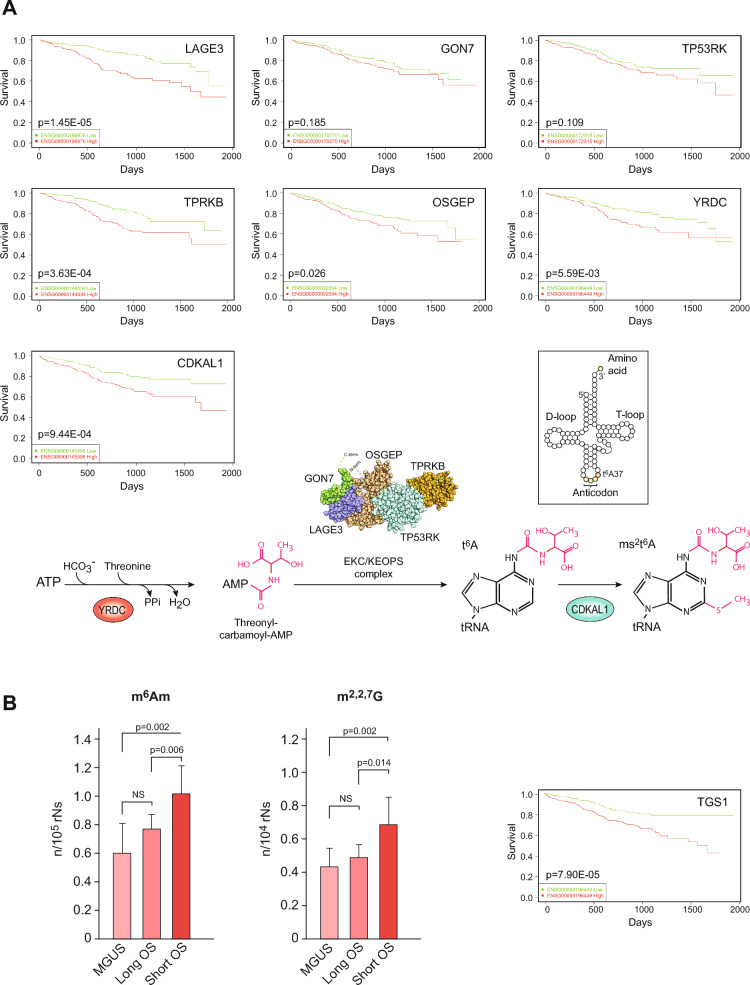
**A** Kaplan–Meier survival plots of patients expressing high (red), or low (green) levels of proteins involved in t^6^A37 modification of tRNAs decoding ANN-codons (patient data from the CoMMpass database). The bottom panel illustrates the reactions involved and a 3D model of the human EKC/KEOPS complex [142] (available via license Creative Commons Attribution 4.0 International). **B** MM patients with short survival after diagnosis had significantly increased levels of m^6^Am and m^2,2,7^G in the total-RNA pool of their malignant cells

The most significantly upregulated protein in MM vs. MGUS was LAGE3 (2.5-fold, p = 6.2E-11). LAGE3 is part of the EKC/KEOPS complex, which deposits N^6^-threonylcarbamoyl at adenosine 37 (t^6^A_37_) of tRNAs decoding ANN codons [42] (Fig. 2). This modification strengthens the interaction of the A-U codon-anticodon base pair in ANN codons, thus enabling proper translation initiation at the AUG start codon as well as preventing frameshifting during translation. Two other subunits of the complex, GON7 and TP53RK, were also among the most significantly upregulated (1.95-fold, p = 1.9E-10 and 1.87-fold, p = 2.9E-9). The final two subunits, OSGEP and TPRKB were also upregulated, although to a lesser extent (1.40-fold, p = 5.8E-5 and 1.35-fold, p = 1.4E-3). We hypothesize that upregulation of the EKC/KEOPS complex contributes to increase translational fidelity in MM cells and that this mediates increased cellular fitness by reducing protein misfolding and aggregation. The latter is substantiated by studies in yeast and *Drosophila* [43] in which t^6^A deficiency is associated with growth deficiency, protein aggregation and UPR induction. In humans, mutations in the ECK/KEOPS complex lead to severe neurological disease and the TP53RK inhibitor fusidic acid has been shown to inhibit colon cancer metastasis to the lung in mice when combined with 5-fluorouracil [44]. Analysis of mRNA expression data from the MMRF CoMMpass study (https://gdc.cancer.gov/about-gdc/contributed-genomic-data-cancer-research/foundation-medicine/multiple-myeloma-research-foundation-mmrf) revealed that high mRNA expression of either *LAGE3*, *TPRKB* or *OSGEP* mediate significantly reduced OS in MM (Fig. 3A). Although high expression of *TP53RK* was only sub-significantly (p = 0.11) associated with reduced OS, a separate study validated TP53RK as a novel therapeutic target in poor-prognosis MM [45]. We also analyzed OS in CoMMpass patients with varying levels of *YRDC*, which encodes the enzyme producing the threonyl-carbamoyl-AMP precursor for t^6^A_37_ deposition, as well as *CDKAL1* that converts t^6^A to ms_2_t^6^A. Although these two enzymes were not quantified in our dataset, high mRNA expression of either mediates significantly reduced OS of MM patients (Fig. 3A). Moreover, analysis of essential genes across pan-cancer based on GeCKO screening data by the DepMap web portal (https://depmap.org/portal/depmap) showed that YRDC gave a lower CERES dependency score (higher dependency) in MM cells than in cells of any other cancer type [46]. We also found a significant increase of the tRNA modifier TRMT6 in MM vs. MGUS (1.5-fold, p = 0.0002). TRMT6 is the substrate-binding subunit of the tRNA N1-adenine methyltransferase, which deposits m^1^A at tRNA position 58. This modification was recently shown to be crucial for enhanced MYC translation in T-cells during their activation [47]. CoMMpass analysis revealed that overexpression of TRMT6 mRNA was associated with reduced OS in MM (p = 0.0025).

These findings prompted us to investigate the other avenues by which RNA modifications may affect translational specificity and fidelity. Of the 226 modified sites in human rRNA identified to date, the most common are 2’-O-methylation at ribose moieties (Nm, 110 sites) and pseudouridylation (ψ, 107 sites) [48]. While many of these are essential to correct rRNA folding, some are also fractionally modified, thus giving rise to dynamic variability and potential re-programming of ribosomes in response to external stimuli. Nm and ψ are directed to rRNA sites by C/D snoRNP- and H/ACA snoRNP complexes, respectively. In these complexes sequence targeting is facilitated by distinct sets of snoRNAs [49, 50] (Fig. 2). Three out of four proteins in the C/D snoRNP were quantified in our dataset and two were significantly upregulated (NOP56; 2.1-fold, p = 9.1E-5, NOP58; 2.1-fold, p = 3.8E-5). Likewise, three out of four proteins in the H/ACA snoRNP complex were significantly upregulated (DKC1; 2.6-fold, p = 4.4E-6, NHP2; 1.9-fold, p = 5.2E-4, GAR1; 1.9-fold, p = 5.9E-4). CoMMpass analysis of mRNAs encoding these snoRNPs revealed that overexpression of all, except NHP2 (p = 0.080), were associated with very significantly reduced OS in MM.

We also found elevated levels of ADAR in MM vs. MGUS (1.6-fold, p = 0.0003). ADAR catalyzes A-to I editing, which is associated with tumorigenesis across several cancers, including MM. A-to-I editing can affect RNA splicing, RNA structure and the function of regulatory RNAs. When occurring in coding sequences, it can also directly alter amino acid incorporation in proteins since inosine is commonly read as guanosine during translation [51]. Hyperactivation of ADAR thus has the potential to rewire the proteome, and promiscuously edited mRNAs could lead to aberrant proteins upon translation. Interestingly, aberrantly edited RNA species may be recognized and withheld in the nucleus, by a protein complex consisting of NONO, SFPQ and MATR3 [52]. These three proteins were all significantly upregulated in MM vs. MGUS.

To explore the potential involvement of epitranscriptomic alterations in MM pathogenesis, we successfully acquired additional biobank samples from ten patients with short OS (mean OS 30 months, mean age 59 years), nine patients with long OS (> 107 months and still alive, mean age 61 years) and 6 MGUS patients (still alive), who were included in the proteome analysis. Unfortunately, the limiting number of cells in the available samples precluded pre-fractionation of RNA into different subspecies prior to LC–MS/MS analysis. This makes it difficult to precisely quantify modifications in specific RNA pools, since many modifications are present in several RNA species. Nevertheless, we found significantly increased levels in total-RNA of N6,2’-O-dimethyladenosine (m^6^Am) and 2,2,7-trimethylguanosine (m^2,2,7^G, TMG) in patients with short vs. long OS (Fig. 3B). m^6^Am is found adjacent to the cap in a significant fraction of vertebrate mRNAs and internally in U2 small nuclear RNAs. Whereas an early study reported that m^6^Am increased mRNA stability, this has later been challenged ([53] and references therein). Rather, this modification appears to suppress cap-dependent translation [54]. Further evaluation of this modification in the context of MM must, however, await identification of m^6^Am readers. Interestingly, 7-meG cap hypermethylation was the top enriched biological process in MM vs. MGUS according to PANTHER GO analysis (Fig. 1C). The TMG cap is found in sn- and snoRNAs, including those of the C/D and H/ACA snoRNPs that mediate pseudouridylation and 2’-O methylation of other RNAs. Trimethylguanosine synthase (TGS1), which catalyzes the two sequential methylation steps of m^7^G was not quantified in our dataset, but CoMMpass analysis reported very significantly reduced OS in MM patients expressing high TGS1 mRNA (Fig. 3B).

In summary, we find that RNA-modifying enzymes that hold the capacity to enhance translational fidelity and rewire the expressed proteome are among the most significantly upregulated in MM compared to MGUS, in agreement with a poor prognosis associated with their mRNA overexpression in MM cells.

### Progression from MGUS to MM is accompanied by downregulated integrin signaling and proteins involved in cell adhesion, motility and rigidity sensing

**Fig. 4:**
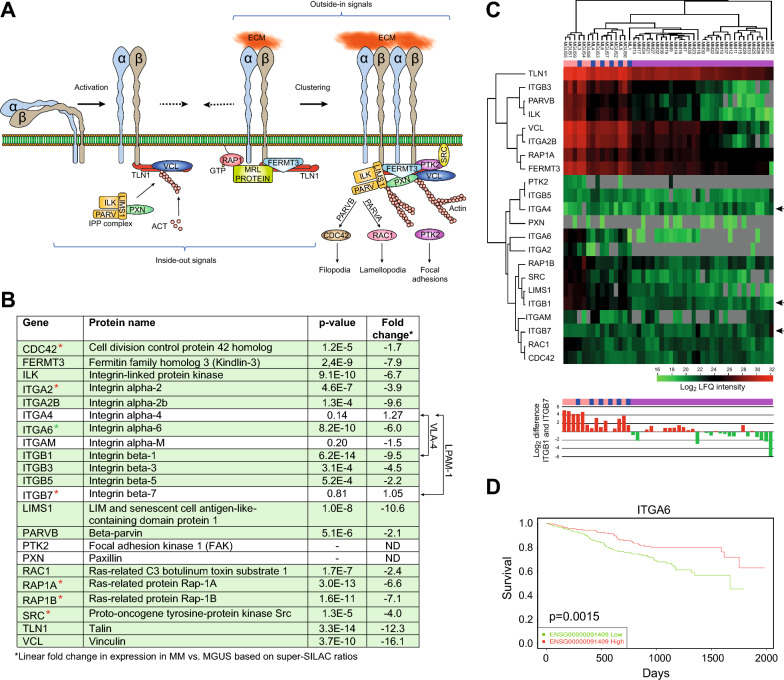
Integrin and Actin cytoskeleton downregulation in MM. **A** Integrin extracellular binding is actively regulated from the inside of the cell and ligand binding to integrins leads to integrin clustering and signaling into the cell. **B** The super-SILAC data show that most proteins involved in these pathways are significantly downregulated in MM vs. MGUS (**C**). Hierarchical clustering of the involved proteins based on their LFQ values (upper panel). Black arrows indicate the three integrins that constitute the VLA-4 and LPAM-1 heterodimers. There is a marked shift from a high- to a low ITGB1/ITGB7 ratio during progression from MGUS to MM (bottom panel). Grey: Not detected. **D** Analysis of MMRF CoMMpass data demonstrate that high ITGA6 mRNA expression is associated with favorable OS in MM

IPA analysis reported Integrin- and Actin cytoskeletal signaling as the two most downregulated pathways in both MM vs. MGUS (Fig. 1D) and MM vs. ML (Fig. 1E). These pathways are highly integrated and involve several common proteins, as illustrated in Fig. 4A. Integrins are heterodimeric transmembrane receptors that facilitate adhesion to the extracellular matrix (ECM) via the so-called integrin-adhesome and are involved in signaling cascades regulating cell growth, motility, survival, rigidity sensing and other responses to the local environment [55]. Upon binding to ECM, integrins cluster and recruit adhesion proteins, including SRC kinase, which then promotes ligand independent activation of EGFR/ERBB2 and activation of the rigidity-sensing apparatus of the cell ([56] and references therein). The core axis of this signaling consists of an integrin heterodimer, talin (TLN) and α-actinin (ACTN). Notably, out of nine integrins quantified in our dataset, six were significantly downregulated in MM compared to MGUS (2.2–9.5-fold), whereas three remained unaffected. Moreover, talin-1 (TLN1) was 12.3-fold downregulated, and α-actinin 1, 2 and 4 (ACTN1/2/4) were 9.6-, 7.6- and 2.6-fold downregulated, respectively. We also found reduced expression of several proteins involved in rigidity sensing in MM compared to MGUS, such as tropomyosin 2.1 (TPM2) [57], which was 6.5-fold downregulated. Finally, we found a fourfold downregulation of SRC kinase and 1.9-fold downregulation of the ERBB2 stabilizer ERBIN.

Altered expression of several integrins have been associated with MM. Among the most extensively studied is VLA-4, which is a heterodimer of ITGA4 and ITGB1 (α4β1). Several studies indicate that increased VLA-4 is associated with increased aggressiveness and drug resistance. Reduced VLA-4 activation has also been attributed to reduced ECM adhesion of MM cells after treatment with the ITGA4 monoclonal antibody natalizumab ([58] and references therein). This does not immediately conform with our findings, since we find a strong and highly significant downregulation of ITGB1 in both MM vs. MGUS and MM vs. ML, whereas ITGA4 remains similar in both groups as well as in ML vs. MGUS (Fig. 4B, Supplementary Table 2). Lack of active VLA-4 in MM is further supported by the strong downregulation of RAP1A and RAP1B (Fig. 4B) since depletion of either Rap1a or Rap1b has previously been shown to impair the activation of VLA-4 on lymphocytes [59, 60]. Conceivably, the downregulation of ITGB1 may facilitate increased binding of ITGA4 to its alternative binding partner ITGB7, which was not significantly differentially regulated between the three disease groups (Fig. 4B, Supplementary Table 2). The ITGA4/B7 receptor (α4β7, LPAM-1) is expressed on a variety of leukocytes, and most studies have addressed its role in chronic inflammatory disorders of the gastrointestinal tract. Here, LPAM-1 mediates homing of immune cells to the addressin MADCAM1 in inflamed gut [61]. LPAM-1 also promotes homing of hematopoietic progenitor cells (HPCs) to the bone marrow and apparently plays a prominent role in the initial tethering and rolling steps but is not required for firm adhesion in the BM microvasculature [62]. High expression of ITGB7 contributes to MM-cell adhesion, migration, invasion, BM homing, and drug resistance [63] and this was supported by CoMMpass analysis, which reported significantly reduced OS in patients expressing high ITGB7 mRNA levels (p = 0.020). This is further supported by a very recent study demonstrating epigenetically enhanced expression of ITGB7 in myelomas with high-risk cytogenetics [64]. Since the super-SILAC data primarily yield information on relative changes in protein amount, we also compared the expression of the proteins in Fig. 4B based on LFQ intensities, which better represent the true protein levels (Fig. 4C, upper panel). This revealed a marked shift in the ratio of LFQ intensities between ITGB1 and ITGB7 from a high ratio in the MGUS and ML groups to a low ratio in the MM group (Fig. 4C, bottom panel). This would conform to a shift from VLA-1 (α4β1) to LPAM-1 (α4β7) during progression to MM. Further contributing to a shift towards active LPAM-1 would be that unlike ITGB1, ITGB7 apparently adopts a constitutive active conformation on MM cells by a yet unknown mechanism [65]. Interestingly, clinical trials are now underway with chimeric antigen receptor T cell (CAR-T) therapy targeting an epitope (MMG49) that is only accessible in activated ITGB7 (NCT04649073), whereas another CAR-T trial targets full-length ITGB7 (NCT03778346). Our results suggest that the ITGB1/ITGB7 ratio could be a promising determinant for the response to such therapies.

Downregulation of *ITGA6* mRNA was recently shown to contribute to the invasion of MM, progression to plasma cell leukemia (PCL) and to reduced OS [66]. This is supported by our data, in which ITGA6 protein was sixfold downregulated in MM vs. MGUS (p = 8.0E-10) and twofold downregulated in MM vs. ML (p = 1.0E-4) but remained unchanged in ML vs. MGUS. This also substantiates that that ITGA6 downregulation is a late step in MM progression. Kaplan-Meyer analysis of CoMMpass data furthermore confirmed that low expression of *ITGA6* mRNA predicted reduced OS (Fig. 4D).

### Progression to MM is associated with overall downregulation of surface antigens

**Fig. 5:**
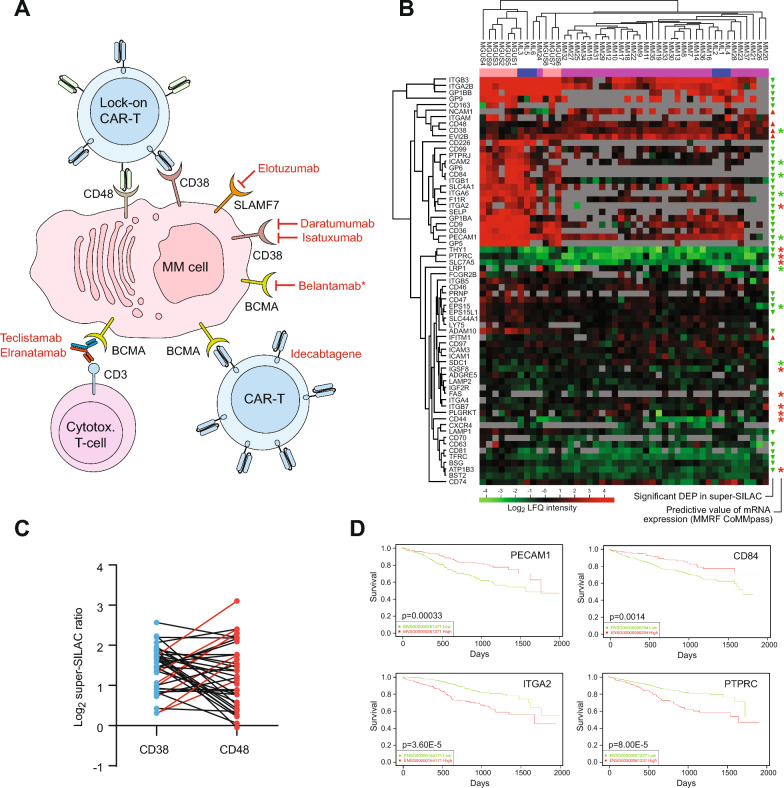
Surface antigens are downregulated in MM vs. MGUS. **A** Immune therapies targeting surface antigens in multiple MM (approved drugs in red. *; withdrawn). **B** Hierarchical clustering of surface antigens in MGUS, ML and MM (peach, blue and violet in top bar, respectively) based on their LFQ intensities. Significantly upregulated (red triangles) and downregulated (green triangles) proteins in MM vs. MGUS (p < 0.05) based on super-SILAC data. Asterisks indicate that the levels of the corresponding mRNAs (MMRF CoMMpass data) may predict good/poor overall survival. Red asterisks: High mRNA expression predicts poor overall survival (p < 0.05). Green asterisks: Low mRNA expression predicts poor overall survival (p < 0.05). Grey: not detected. **C** Both CD38 and CD48 expression varied considerably among the patients. Whereas low CD38 alone would signify poor response to Daratumumab, a low CD38/CD45 ratio (red connecting lines) could signify poor response to CD38/CD48 “lock-on CAR-T therapy. In both instances, co-treatment with ATRA could be beneficial to increase CD38 expression. **D** Analysis of MMRF CoMMpass data revealed that several of the surface antigens harbored prognostic information when analyzed at the mRNA level. Examples of positive prognosis when overexpressed are PECAM1 and CD84, whereas ITGA2 and PTPRC were negatively prognostic

Surface antigens mediate communication of MM cells with the BM and are crucial determinants for the response of malignant cells to immunotherapy. The currently approved therapies are the monoclonal antibodies Daratumumab and Isatuximab that target CD38 and Elotuzumab that targets SLAMF7. Belantamab (conjugated to the cytotoxic drug monomethyl auristatin F) that targets TNFRSF17 (BCMA) [67], is currently under investigation in several clinical studies of combination regimens. Two CAR-T therapies, Ciltacabtagene [68] and Idecabtagene [69] also target BCMA, and have been approved for subsets of patients with advanced MM. Finally, the recently approved bispecific antibodies (BsAbs) Teclistamab and Elranatamab target both BCMA on MM cells and the T-cell antigen CD3 [70, 71] (Fig. 5A). Of the 65 verified surface antigens quantified in our study (MHC not included), 41 were found to be significantly differentially expressed (p < 0.05) in MM vs. MGUS. Among these, 36 were downregulated, whereas five, CD38, CD48, EVI2B (CD361), IFITM1 (CD225) and NCAM1 were upregulated (Fig. 5B). CD38 was also among the highest expressed antigens across all MM samples, corroborating the effect of anti-CD38 therapies. A recent study proposed additional targeting of CD48 as a potential high copy number target in in a recently suggested “lock-on” CAR-T approach [72] (Fig. 5A). Although CD48 was readily quantified in all MM samples, it displayed somewhat lower and more variable expression than CD38, potentially warranting careful patient stratification prior to such therapy. For patients with low CD38 expression or a low CD38/CD48 ratio (Fig. 5C), all-trans retinoic acid (ATRA) could be beneficial to enhance CD38 expression [73] and improve responses to Daratumumab or CD38/CD45 “lock-on” CAR-T therapy, respectively. Interestingly, a recent pan-cancer plasma proteomics study employing the Olink^®^ Proximity Extension Assay reported CD38 and CD48 among the 12 most upregulated proteins in plasma from myeloma patients compared to plasma from other cancer patients (https://insight.olink.com/data-stories/disease-atlas-cancer), highlighting their potential as non-invasive biomarkers to aid treatment decisions.

IFITM1 is an interferon-induced antigen that inhibits entry of several viruses into the host cytoplasm and predicts poor prognosis across several cancers [74]. IFITM1 mRNA expression is increased in MM cells compared to healthy plasma cells [75], but its potential significance in MM pathogenesis remains to be investigated. Neural cell adhesion molecule 1 (NCAM1, CD56) was threefold upregulated in MM vs. MGUS. It is a membrane glycoprotein and belongs to the immunoglobulin superfamily and is involved in cell-to-cell interactions as well as cell–matrix interactions during development and differentiation. A study using immunohistochemistry showed that there is a significant correlation between strong expression of NCAM1 by MM cells and the presence of lytic bone lesions. In agreement with our findings, the study concluded that NCAM1 expression to be a reliable diagnostic criterion in distinguishing MM from MGUS and other plasma cell disorders [76]. CD56 was included in a recent multitarget CAR-T clinical study (NCT03271632), but the results have not yet been published.

It is tempting to speculate that the overall downregulation of surface antigens may confer a survival advantage to myeloma cells by allowing them to escape detection by the immune system and to resist immunotherapy. The most significantly downregulated was CD84 (SLAM5) (p = 3.94E-13), which was readily detected in eight out of nine MGUS samples, two out of six ML, but in neither of the 31 MM samples. CD84 is a self-ligand receptor of the signaling lymphocytic activation molecule (SLAM) family. It has previously received little attention in MM but has been suggested to regulate a survival pathway in chronic lymphocytic leukemia (CLL), by bridging to cells in the microenvironment. A very recent study demonstrated that MM cells express low or undetectable levels of CD84 [77], in agreement with our results. However, they found no significant difference in surface CD84 expression on CD138 + cells from MGUS, SMM or MM. The reason for the latter discrepancy is unclear but may be caused by few MGUS patients (n = 3) in the previous study. Interestingly, the study revealed a very significant increase in CD84 on BM stroma cells from MM patients compared to healthy donors. Apparently, this is induced by macrophage migration inhibitory factor (MIF) excreted from the MM cells, and the authors propose that MIF-induced CD84 regulates PD-L1/PD-1 and exhaustion marker expression on MDSCs and T cells, respectively, resulting in a downregulated immune response [77]. We observed a 1.8-fold (p = 9.2E-5) increased MIF expression in MM vs. MGUS, supporting further evaluation of CD84 as a novel drug target in MM.

Many of the quantified CD antigens that were differentially expressed in MM vs. MGUS have previously not been associated with MM. One such example is the CD42 complex (GPIb-V-IX-complex). It consists of GP9, GP1BA, GP1BB and GP5 (CD42A-D, respectively), which were 5.3–19.9-fold downregulated in MM vs. MGUS (Fig. 5B). CD42 is highly expressed on platelets. When blood vessels are damaged, von Willebrand factor (VWF) in plasma binds to collagen in the exposed ECM. VWF then undergoes a conformational change, binds to CD42 and arrests platelets to form a plug at sites of vascular injury [78]. Although platelets have been shown to play pivotal functions in progression and metastasis of many cancers [79], the marked downregulation of CD42 complex in MM cells is not immediately reconciled with increased proliferative or metastatic potential. Neither of the subunits yielded significant prognostic information by analysis of CoMMpass mRNA data, but low expression of GP1BB (CD42C) has been associated with poor prognosis in osteosarcoma [80]. Potentially, downregulation of CD42 in MM might moonlight altered exchange of surface proteins between platelets and the malignant cells. Such exchange was recently described in two independent studies, where CD42 was hijacked from platelets by phagocytosis and recycled to the surface membrane of several cancer cell lines [81, 82]. If this holds true, it implies that phagocytic uptake of platelets and/or the degradation of phagocytic components, is reduced in MM vs. MGUS. In support of this, the two most downregulated GO biological processes reported from the common DEPs in MM vs. MGUS and MM vs. ML were Protein secretion by platelet and Positive regulation of protein processing in phagocytic vesicle (Fig. 1C). The molecular mechanisms underlying the apparently contradictory results from the in vitro studies [81, 82] and the present study clearly warrant further investigation. This also holds true for the role of RAB-GTPases in the regulation of surface proteins in MM. This family of small GTPases play a critical role in endosomal membrane trafficking as well as transport of proteins to and from the cell surface and are often dysregulated in cancer [83]. Among the 29 RAB-GTPases quantified in our study, 19 were significantly downregulated (p < 0.05) and one (RAB43) was significantly upregulated (Supplementary Table 2). RAB43 did not yield any prognostic information from CoMMpass analysis, but its upregulation has been shown to promote cell adhesion and invasion in gliomas [84].

Analysis of CoMMpass data revealed that for five of the antigens that were downregulated in MM vs. MGUS (CD84, EPS15, ICAM2, PECAM1, ITGA6) low mRNA expression also predicted poor OS (Figs. 4D and 5B, D). Downregulation of PECAM1 was also associated with poor prognosis in MM in a very recent study from another group [85]. This downregulation was attributed to enhanced expression of the transcription regulator E2F2 and led to cell migration-mediated MM progression by inhibiting cell adhesion. Somewhat surprisingly, we found significant downregulation of three proteins (PTPRC, THY1, ITGA2) that are positively correlated with OS when downregulated at the at the mRNA level (Fig. 5B, D). PTPRC (CD45), previously known as leukocyte common antigen, is a receptor protein tyrosine phosphatase that plays a critical role in antigen receptor signaling and lymphocyte development [86]. PTPRC was significantly downregulated in MM vs. MGUS (threefold, p = 8.5E-6) as well as in MM vs. ML (twofold, p = 0.01), in agreement with a previous study that demonstrated progressive decline of the receptor during progression of MM [87]. This may be caused by a clonal shift in the MM population during progression. In the initial phase, CD45^+^ MM cells dominate, which have a high capacity of homing to the bone marrow and can grow in response to IL6. However, they are also more susceptible to apoptosis upon stimulation, whereas the more slowly growing CD45^−^ cells are more resilient to apoptosis and gradually dominate [[88] and references therein]. In agreement with this, a very recent mass cytometry study [89] identified a MM subgroup with favorable treatment response and improved OS, which was characterized by elevated PTPRC and reduced expression of anti-apoptotic BCL2 (our data revealed twofold increased BCL2 in MM vs. MGUS, p = 0.007). Notwithstanding the role of PTPRC in disease progression, its expression is linked to drug response since CD45^−^ cells are more sensitive to inhibitors targeting AKT/PI3K signaling [90]. THY1 (CD90) is a surface glycoprotein that interacts with integrins in *cis* and *trans* and is upregulated in myeloma (proteinatlas.org), but little known about its relevance to disease progression. It has tumor-promoting or -suppressing effects depending on cell type, and its tumor-suppressor activity appears to be dependent on ITGB3 interaction [91]. ITGB3 was 4.5-fold downregulated in MM vs. MGUS (p = 3.1E-4) and thus less able to exert such a function. Interestingly, bortezomib upregulates ITGB3 expression in MM cells [92] and targeting of THY1 in synergy with bortezomib should thus be evaluated in MM treatment. Finally, in addition to the surface antigens shown in Fig. 5B, we observed an overall downregulation of proteins involved in antigen processing and presentation. Both via MHC I (HLA-A and -B) and MHC II (HLA-DQA1, -DRA and -DRB1) (Supplementary Table 2), suggesting reduced recognition by CD4^+^/CD8^+^ T-cells.

Taken together, progression from MGUS to MM is accompanied by an overall reduced expression of surface antigens that may contribute to immune evasion of the malignant cells and modulate interaction with the bone marrow microenvironment. It is also tempting to speculate that the reduced load of proteins entering the folding and modification apparatus of the ER might contribute to reduced protein aggregation and hinder induction of the unfolded protein response (UPR). The latter is supported by the significant upregulation of the co-chaperones DNAJC1 (ERdj1), SEC63 (ERdj2) and SEC62 (Supplementary Table 2) that interact with ribosomes at the ER to suppress translation and translocation into ER and thus avoid induction of the UPR [93].

### DNA repair and genome maintenance proteins are upregulated in MM compared to MGUS

**Fig. 6:**
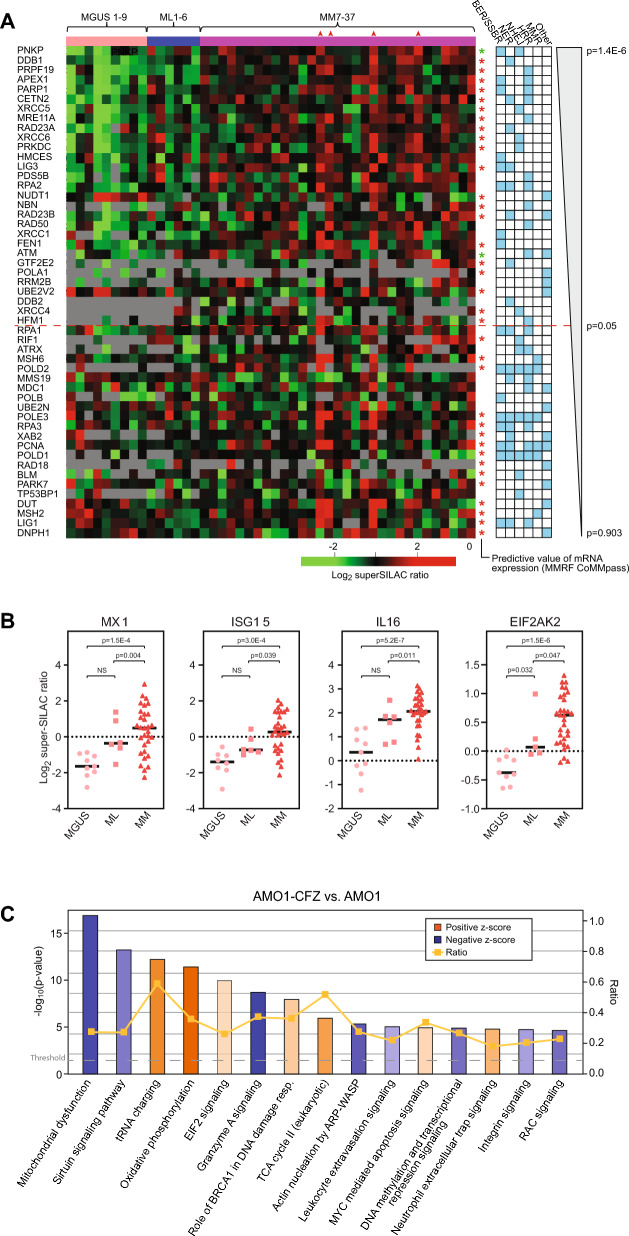
**A** DNA repair and genome maintenance proteins quantified in our study and ranged with respect to increasing p-value (MM vs. MGUS). DNA repair pathways in which the proteins exert their major functions are highlighted as light blue squares. Asterisks indicate that the levels of the corresponding mRNAs (MMRF CoMMpass data) may predict good/poor overall survival. Red asterisks: High mRNA expression predicts poor overall survival (p < 0.05). Green asterisks: Low mRNA expression predicts poor overall survival (p < 0.05). *BER* Base excision repair, *HRR* Homologous recombination repair, *NHEJ* Non-homologous end-joining, *MMR* Mismatch repair, *SSBR* Single-strand break repair. **B** Several interferon-stimulated proteins were significantly upregulated during progression from MGUS to MM. **C** IPA pathway analysis of carfilzomib-resistant vs. -sensitive AMO1 MM cells revealed a pattern of affected pathways resembling those observed in MM vs. MGUS (Fig. 1D), corroborating the importance of maintaining proteostasis in MM cells in vivo

Appropriate regulation of DNA repair is critical to successful immunoglobulin VDJ recombination, somatic hypermutation (SHM) and class switch recombination (CSR) in the early stages of plasma cell development [94, 95]. Since DNA damaging agents are widely used in the treatment of MM [96], it is also not surprising that the expression levels of DNA repair proteins can predict responses to such agents [97, 98]. We thus manually interrogated our data to evaluate potentially altered genome maintenance pathways. Out of 220 reported DNA repair and DNA damage response proteins [99], 51 were quantified in our dataset. Strikingly, 28 of these were significantly upregulated, whereas two (NUDT1 and UBE2V2) were downregulated (Fig. 6A). Moreover, among the upregulated proteins, 20 also predicted poor OS when highly expressed at the mRNA level. Somewhat unexpectedly, PNKP, which was the most significantly upregulated (twofold, p = 1.4E-6) predicted good OS when overexpressed at the mRNA level. The underlying reason for this remains elusive, since PNKP is necessary to process DNA ends by its 3’-phosphatase and 5’-kinase activities in both base excision-/single strand break repair (BER/SSBR) and in non-homologous end-joining (NHEJ). Potentially PNKP activity needs to be maintained within a specific window to avoid cytotoxic effects. This is also supported by its extensive post-translational regulation, which involves multiple mono-ubiquitnylations by CUL4-DDB1-STRAP and double phosphorylation by the PI3K-like kinase ATM [100]. High ATM mRNA expression also predicted good OS, even if it was 1.7-fold upregulated in MM vs. MGUS at the protein level (p = 0.013). This would seem counterintuitive given its central role in the cellular response to DSBs by phosphorylating several downstream targets that orchestrate HRR and cell-cycle arrest [101]. However, previous studies, including studies from our own laboratory, suggest that MM cells do not rely on ATM for repair of chemotherapy-induced DNA lesions, even if it is apparently activated by DNA damage in MM cells [97, 102].

Whereas the upregulated proteins could be assigned to several DNA repair pathways, repair of DSBs via homologous recombination repair (HRR) or NHEJ was clearly over-represented. There were also some patients that displayed overall upregulation of proteins belonging to several repair pathways (illustrated by red arrows in Fig. 6A). We speculate that this may reflect higher MM cell proliferation in these patients. Many DNA repair proteins are cell cycle regulated, with highest expression in S-phase [103] and the indicated samples also display high expression of the proliferation markers PCNA (Fig. 6A) and MKI67 (Supplementary Table 2). Potentially, these patients belong to a proliferation (PR) subgroup previously identified by gene expression profiling and with especially poor prognosis [104]. The MM cells in this group may also be subject to replicative stress, a common feature of cells overexpressing MYC, and thus be sensitive to ATR inhibition, especially when co-administered with DNA-damaging agents [105]. Although ATR was not quantified in our data, this is supported by previous studies in our group, in which the MM cell line RPMI8226 (classified as “high damage” cells [105]) harboring acquired resistance to melphalan, was exquisitely sensitive to the ATR inhibitor VE-821 [97]. Four ATR inhibitors (berzosertib, ceralasertib, elimusertib and gartisertib) are currently in clinical trials against a variety of solid tumors, but surprisingly few trials encompass hematological malignancies ([106] and references therein). Noteworthy, however, several of the trials involve co-treatment with PARP1 inhibitors, under the presumption that this would inhibit SSBR and increase replication stress. PARP1 was 1.9-fold (p = 1.3E-5) upregulated in MM vs. MGUS and is involved in a multitude of cellular processes. In cancer treatment, PARP inhibitors are particularly effective against BRCA1/2-mutant tumors [107]. Here, they inhibit repair of DNA SSBs, which are then converted to DSBs during replication. In BRCA1/2-defective tumors these DSBs cannot be corrected by HRR, leading to synthetic lethality of the tumor cells. BRCA1/2-mediated HRR-deficiency appears to be rare in MM [108]. The latter conforms to our findings, suggesting that HRR is overall upregulated in MM vs. MGUS, at least based on the expression levels of the proteins involved. Nevertheless, several recent studies in MM cell lines and xenograft models have demonstrated that PARP1 expression and/or activity in MM is inversely correlated with sensitivity to chemotherapeutic agents, including melphalan [109], bortezomib [110] and temozolomide [111]. This is also underscored by Kaplan–Meier analysis demonstrating that high expression PARP1 mRNA is associated with significantly shortened OS (p = 0.001). Potential effects of PARP1-inhibitors against MM in the clinical setting remain elusive. One phase 1 study encompassing a limited number of B-cell lymphomas and one MM, investigated co-treatment with the PARP1-inhibitor veliparib and bendamustine/rituximab. The treatment was overall well tolerated and objective responses were reported [112]. Based on the above, clinical studies of both PARP1- and ATR-inhibitors in MM are highly warranted.

In addition to the proteins shown in Fig. 6A, many proteins may influence DNA repair efficiency and genome stability indirectly, e.g., by modulating chromatin status. This includes high mobility group (HMG) proteins, which constitute the second most abundant chromatin proteins after histones and are critical to regulate gene expression and DNA repair [113]. Of the eight HMGs quantified in our dataset, five (HMGA1, HMGB3, HMGN1, HMGN2 and HMGN3 were significantly upregulated (1.9–2.7-fold) in MM vs. MGUS. HMGA1 binds to and is a substrate for DNA-PK (PRKDC), and stimulates LIG4 activity, thus establishing a role in NHEJ [114]. HMGA1 is also phosphorylated by ATM [115], thereby stimulating the transcriptional activity of the ATM promoter [116] in agreement with the significantly increased ATM protein expression in MM vs. MGUS. HMGB3 is highly expressed in a variety of cancers and is associated with tumor proliferation and drug resistance [117]. It binds intrastrand cisplatin crosslinks in DNA [118] and inhibition of HMGB3 in cisplatin-resistant ovarian cancer cells attenuated the ATR/CHK1 DNA damage signaling pathway [119]. HMGN1 may promote base excision repair via activation of PARP1 [120] and DSB repair via promoting activation of ATM [121]. In agreement with the roles of the HMG proteins in DNA repair and chemoresistance, Kaplan–Meier analysis of CoMMpass data demonstrated that enhanced mRNA expression of HMGA1 (p = 0.033), HMGB3 (p = 1.67E-13), HMGN1 (p = 0.016) and HMGN2 (p = 0.0004) was associated with significantly decreased OS.

In addition to the obvious role of DNA repair proteins in the response to DNA-damaging chemotherapeutic agents, increased overall DNA repair capacity in MM vs. MGUS likely contributes to increased translational fidelity by reducing mutational burden. Missense mutations, indels and translocations may all result in aberrant and misfolded proteins that mediate proteotoxic stress. Importantly, even synonymous mutations may profoundly affect the final protein structure since correct folding depends on translation rate and the latter is dictated by the availability of the relevant isoacceptor tRNAs in the cell [122].

### Interferon-stimulated proteins are upregulated in MM vs. MGUS

Many interferon-stimulated genes (ISGs) such as MX1 (3.8-fold), ISG15 (3.0-fold) and IL16 (3.3-fold) were among the most upregulated in MM vs. MGUS (Fig. 6B). Several of the upregulated ISGs also belong to a subgroup known as IFN-related DNA damage resistance signature (IRDS) and that is positively correlated with therapy resistance across multiple cancer types [123]. Upstream regulators of the IRDS include EIF2AK2 and STAT1, that were both significantly upregulated in MM vs. MGUS (Fig. 6B, Supplementary Table 2). Since no interferons or interferon receptors were quantified in our dataset, it remains unclear whether this upregulation has an autocrine or paracrine origin. However, interferons may be induced via the cGAS-STING pathway. Recently, UNC13D was found to inhibit the synthesis of interferons and inflammatory cytokines via this pathway by repressing oligomerization of STING1 at the ER [124]. UNC13D was 4.7-fold downregulated in MM vs. MGUS (p = 0.0002), potentially contributing to ISG activation.

Several studies have addressed the effects of IFN-α treatment in MM, but the results are conflicting and low response rates (10–25%) have been observed [125]. In agreement with this, CoMMpass analysis revealed no significant association between MX1, ISG15 and IL16 mRNA levels with OS. Nevertheless, it cannot be excluded that low levels of IFN-I-stimulated proteins could serve as a biomarker for patients that would benefit from IFN-α maintenance therapy [125]. Paradoxically, MX1 has been found to inhibit motility in prostate carcinoma cells and thereby to inhibit metastasis [126]. However, MX1 and other interferon-stimulated genes (ISGs) apparently mediate differential effects in different cancer cell types. Thus, unfavorable effects have been observed in aromatase inhibitor-resistant breast cancer [127] and in the node-negative ER + /ERBB2- subtype [128].

IFN stimulation has been shown to mediate a shift in the cellular proteasome composition, towards increased formation of immunoproteasomes. This was also supported from our proteomic data. Only one of the 14 α/β subunits of the canonical 20S core proteasome was significantly differentially expressed in MM vs. MGUS (PSMB6, 1.5-fold downregulated, p = 0.007). In the immunoproteasome, three of the β subunits, PSMB5/6/7 (β5/β1/β2) are replaced by PSMB8/9/10 (β5i/β1i/β2i) and of these, PSMB9 and 10 were both upregulated in MM vs. MGUS (1.3-fold, p = 0.004 and p = 0.03, respectively). Although the downregulation of β1 and upregulation β1i and β2i appears modest, formation of immunoproteasomes is likely additionally favored by their approximately four times faster assembly rate compared to conventional 20S proteasomes [129]. In addition to its role in generating peptides to be presented by MHC class I molecules, the immunoproteasome contributes to clearance of oxidized proteins and thus maintaining proteostasis [130]. The activity and selectivity for oxidized proteins is enhanced by binding of the trimeric 11S (Pa28) regulator [131]. In support of such a function in MM pathogenesis, we find significantly increased levels of all three 11S subunits, most notably PSME1 (1.5-fold, p = 7.5E-6) and PSME2 (1.7-fold, p = 1.4E-6) in MM vs. MGUS. Both proteins were also significantly increased in ML vs. MGUS.

Interleukin-16 (IL16) is a recently discovered biomarker in MM. MM cells constitutively express IL16 and spontaneously secrete the soluble form of the cytokine as an autocrine factor that promotes MM cell proliferation. Elevated levels of IL16 have been found in peripheral blood of MM patients and increase with disease progression [132, 133]. A recent study also demonstrated that IL16 stimulates osteoclast activation and causes monocytes to differentiate into osteoclast-like cells [134]. Inhibition of IL16 could thus represent a new strategy to prevent MM-associated bone loss.

In summary, we find an overall enhancement of the IFN response associated with progression from MGUS to MM. However, the expression of ISGs varied considerably between patients, and may explain previous conflicting and low response rates to IFN treatment. Nevertheless, the resulting switch towards immunoproteasome formation may provide a survival benefit by maintaining proteostasis in the oxidizing inflammatory niche mediated by IFN stimulation.

### Differentially regulated processes in MM vs. MGUS are recapitulated in PI resistant MM cells

Proteasomal mutations, particularly in PSMB5, were initially believed to play a major role in conferring resistance to PIs [135], but subsequent studies have largely failed to identify such mutations in cells from MM patients [136]. An alternative model suggests that PI resistance is associated with diminished activation of the UPR [137]. This was also supported by proteome analysis of AMO1 MM cell lines resistant to either bortezomib or carfilzomib. These analyses revealed a “IRE1/XBP1-low” pattern of UPR activation in the resistant cells, irrespective of proteasome mutations [138]. To further investigate whether the proteome alterations of PI-resistant MM cells resembled those observed in the progression from MGUS to MM, we performed label-free quantification of proteins from carfilzomib sensitive AMO1 cells and their resistant counterparts (AMO1-CFZ). This comprehensive analysis identified more than 2800 DEPs (q-value < 0.05) in the PI-resistant cells (Supplementary Table 3). While our results corroborate many of the findings from the previous study [138], including robust upregulation of the multidrug transporter ABCB1 in AMO1-CFZ, we also unveiled a broad spectrum of novel proteins associated with PI resistance. Remarkably, when we applied IPA^®^ analysis to our data, EIF2-signaling was among the top upregulated biological processes like we observed in MM vs. MGUS. Likewise, MYC was reported as the top upstream activator (z-score 3.9, p = 1.0E-31). Whereas MYC was below the detection level in CFZ sensitive cells, it was robustly detected in AMO1-CFZ (Supplementary Table 3). Furthermore, akin to the MM vs. MGUS comparison, integrin- and RAC-signaling were among the most significantly downregulated biological processes in AMO1-CFZ (Figs. 1D and 6C), corroborating our hypothesis that these pathways are implicated in maintaining proteostasis in MM cells. We also observed significant downregulation of the UPR inducer IRE1 (ERN1) in AMO1-CFZ, in agreement with the model proposing that a low IRE1/XBP1 pattern mediates PI resistance [137, 138]. Notably, the constant regions of the immunoglobulin heavy (IGHA1) and light (IGKC) chains produced by the AMO1 cell line (https://www.cellosaurus.org/CVCL_1806), were 787-fold and 7400-fold downregulated, respectively, in the CFZ-resistant cells. This is entirely in accordance with the reduced immunoglobulin production observed in MM vs. MGUS, and likely contributes significantly to inhibit UPR induction.

IPA analysis reported downregulated mitochondrial dysfunction in AMO1-CFZ (z-score -3.92), while oxidative phosphorylation was upregulated (z-score 5.60) (Fig. 6C). Moreover, 59 of the 89 upregulated ribosomal subunits in AMO1-CFZ were mitochondrial (MRPs) and among the five heat-shock chaperones that were upregulated in AMO1-CFZ, three constitute the mitochondrial HSP family: HSPA9 (mtHSP70, mortalin), HSPD1 (mtHSP60) and HSPE1 (mtHSP10). An important role of mitochondria in maintaining proteostasis in PI-resistant MM cells was further validated by very recent analysis of genome-wide CRISPR-knockout screening data from the Cancer Dependency Map (DepMap) [139]. The authors employed a “co-dependency” approach and identified several cytosolic HSP70 homologs as prime candidates for targeting PI resistance. When a series of allosteric HSP70 inhibitors referred to as "JG" inhibitors were employed, 15 out of 16 compounds demonstrated lower LC50 values in PI-resistant AMO1 cells than in WT cells [140]. However, intriguingly, some of these JG inhibitors primarily targeted mitochondria, primarily HSPA9, leading to reduced levels of MRPs. Moreover, an analysis of CoMMpass data revealed a strong correlation between high HSPA9 expression and shorter OS compared to other HSP70 isoforms, and even shorter Progression-Free Survival (PFS) compared to any of the proteasomal subunits [140]. Based on their findings, the authors propose that assessing the baseline expression levels of HSPA9, and possibly other chaperones, could hold significance in predicting the response to initial PI therapy. While our analysis did not reveal a statistically significant difference in HSPA9 expression between our MGUS and MM patient samples, it is worth noting that there was substantial (~ four-fold) variability observed among individual MM patients (Supplementary Table 2). This intriguing observation prompts the need for a more comprehensive investigation into the extent to which variations in the baseline expression of HSPA9 and other HSP70 isoforms may serve as predictive indicators for responses to upfront PI treatment.

## Conclusions

To the best of our knowledge, our study constitutes the most comprehensive exploration of proteomic alterations that occur as premalignant plasma cells progress from MGUS to malignant MM. During the early stages of malignant transformation, plasma cells apparently employ a multifaceted strategy to bolster proteostasis while evading UPR induction. This includes reducing monoclonal Ig expression to minimize ER stress and elevating ribosomal biogenesis to enhance translational fidelity. As the disease progresses, we observe an increasingly robust DNA repair capacity and heightened expression of epitranscriptomic modifiers, both potentially contributing to enhanced translational fidelity. In the later stages of progression, a distinct pattern emerges, characterized by reduced integrin signaling and diminished surface antigen expression. These changes may serve a dual purpose: preventing UPR induction and facilitating evasion from immune recognition. Significantly, our analysis of Proteasome Inhibitor (PI)-resistant MM cells reinforces many of these findings in patients, underscoring the pivotal role these processes play in preserving cellular proteostasis and resisting treatment with proteasome inhibitors. Our study has uncovered a range of potential novel biomarkers that merit in-depth evaluation of their prognostic utility and their potential to inform treatment decisions. Several of these also constitute potential targets to overcome PI resistance.

### Supplementary Information

Supplementary Material1 (PDF 1322 KB)

Supplementary Material2 (PDF 328 KB)

Supplementary Material3 (PDF 343 KB)

Supplementary Material4 (XLSX 28 KB)

Supplementary Material5 (XLSX 3186 KB)

Supplementary Material6 (XLSX 2349 KB)

## Data Availability

The super-SILAC and LFQ proteomics data have been deposited to the ProteomeXchange Consortium via the PRIDE [141] partner repository with the identifiers PXD006458 and PXD045439, respectively.

## Abbreviations

ASCT: Autologous stem-cell transplantation
BM: Bone marrow
BsAb: Bispecific antibody
CAR-T: Chimeric antigen receptor T cell
CFZ: Carfilzomib
CRAB: Increased calcium, renal insufficiency, anemia, or bone lesions
CSR: Class switch recombination
DDA: Data dependent aquisition
DEP: Differentially expressed protein
DSB: DNA double-strand break
ECM: Extracellular matrix
FBS: Fetal bovine serum
FDR: False discovery rate
GO: Gene ontology
HMG: High mobility group
HPC: Hematopoietic progenitor cell
HRR: Homologous recombination repair
ISG: Interferon-stimulated gene
LFQ: Label-free quantitation
MCP: Monoclonal protein
ML: Multiple myeloma with a MGUS-like proteome profile
MHC: Major histocompatibility complex
MM: Multiple myeloma
MMRF: Multiple Myeloma Research Foundation
MNC: Mononuclear cell
MGUS: Monoclonal gammopathy of undetermined significance
MRP: Mitochondrial ribosomal protein
NHEJ: Non-homologous end-joining
OS: Overall survival
PCL: Plasma cell leukemia
sFLC: Serum free light chain (kappa/lambda) ratio
PI: Proteasome inhibitor
SHM: Somatic hypermutation
SILAC: Stable isotope labeling with amino acids in cell culture
snoRNP: Small nucleolar ribonucleoprotein
TMG: 2,2,7-Trimethylguanosine
UPR: Unfolded protein response

## References

[1] Sung H, Ferlay J, Siegel RL, Laversanne M, Soerjomataram I, Jemal A (2021). Global cancer statistics 2020: GLOBOCAN estimates of incidence and mortality worldwide for 36 cancers in 185 countries. CA Cancer J Clin.

[2] Zhou L, Yu Q, Wei G, Wang L, Huang Y, Hu K (2021). Measuring the global, regional, and national burden of multiple myeloma from 1990 to 2019. BMC Cancer.

[3] van de Donk N, Pawlyn C, Yong KL (2021). Multiple myeloma. Lancet.

[4] Kaur J, Valisekka SS, Hameed M, Bandi PS, Varma S, Onwughalu CJ (2023). Monoclonal gammopathy of undetermined significance: a comprehensive review. Clin Lymphoma Myeloma Leuk.

[5] Kyle RA, Larson DR, Therneau TM, Dispenzieri A, Kumar S, Cerhan JR (2018). Long-term follow-up of monoclonal gammopathy of undetermined significance. N Engl J Med.

[6] Dhodapkar MV, Sexton R, Waheed S, Usmani S, Papanikolaou X, Nair B (2014). Clinical, genomic, and imaging predictors of myeloma progression from asymptomatic monoclonal gammopathies (SWOG S0120). Blood.

[7] Visram A, Soof C, Rajkumar SV, Kumar SK, Bujarski S, Spektor TM (2021). Serum BCMA levels predict outcomes in MGUS and smoldering myeloma patients. Blood Cancer J.

[8] Pérez-Persona E, Vidriales M-B, Mateo G, García-Sanz R, Mateos M-V, de Coca AG (2007). New criteria to identify risk of progression in monoclonal gammopathy of uncertain significance and smoldering multiple myeloma based on multiparameter flow cytometry analysis of bone marrow plasma cells. Blood.

[9] Zhan F, Barlogie B, Arzoumanian V, Huang Y, Williams DR, Hollmig K (2007). Gene-expression signature of benign monoclonal gammopathy evident in multiple myeloma is linked to good prognosis. Blood.

[10] Zhan F, Hardin J, Kordsmeier B, Bumm K, Zheng M, Tian E (2002). Global gene expression profiling of multiple myeloma, monoclonal gammopathy of undetermined significance, and normal bone marrow plasma cells. Blood.

[11] Anguiano A, Tuchman SA, Acharya C, Salter K, Gasparetto C, Zhan F (2009). Gene expression profiles of tumor biology provide a novel approach to prognosis and may guide the selection of therapeutic targets in multiple myeloma. J Clin Oncol.

[12] López-Corral L, Corchete LA, Sarasquete ME, Mateos MV, García-Sanz R, Fermiñán E (2014). Transcriptome analysis reveals molecular profiles associated with evolving steps of monoclonal gammopathies. Haematologica.

[13] Vogel C, Marcotte EM (2012). Insights into the regulation of protein abundance from proteomic and transcriptomic analyses. Nat Rev Genet.

[14] Chanukuppa V, Paul D, Taunk K, Chatterjee T, Sharma S, Shirolkar A (2020). Proteomics and functional study reveal marginal zone B and B1 cell specific protein as a candidate marker of multiple myeloma. Int J Oncol.

[15] Wu X, Guo J, Chen Y, Liu X, Yang G, Wu Y (2020). The 60-kDa heat shock protein regulates energy rearrangement and protein synthesis to promote proliferation of multiple myeloma cells. Br J Haematol.

[16] Ferguson ID, Patino-Escobar B, Tuomivaara ST, Lin YT, Nix MA, Leung KK (2022). The surfaceome of multiple myeloma cells suggests potential immunotherapeutic strategies and protein markers of drug resistance. Nat Commun.

[17] Giliberto M, Santana LM, Holien T, Misund K, Nakken S, Vodak D (2022). Mutational analysis and protein profiling predict drug sensitivity in multiple myeloma cell lines. Front Oncol.

[18] Yao L, Wang JT, Jayasinghe RG, O'Neal J, Tsai CF, Rettig MP (2023). Single-cell discovery and multi-omic characterization of therapeutic targets in multiple myeloma. Cancer Res.

[19] Ong S-E, Blagoev B, Kratchmarova I, Kristensen DB, Steen H, Pandey A (2002). Stable isotope labeling by amino acids in cell culture, SILAC, as a simple and accurate approach to expression proteomics. Mol Cell Proteomics.

[20] Geiger T, Cox J, Ostasiewicz P, Wisniewski JR, Mann M (2010). Super-SILAC mix for quantitative proteomics of human tumor tissue. Nat Methods.

[21] Zatula A, Dikic A, Mulder C, Sharma A, Vågbø CB, Sousa MML (2017). Proteome alterations associated with transformation of multiple myeloma to secondary plasma cell leukemia. Oncotarget.

[22] International Myeloma Working G (2003). Criteria for the classification of monoclonal gammopathies, multiple myeloma and related disorders: a report of the international myeloma working group. Br J Haematol.

[23] Roseth Aass K, Nedal TMV, Anshushaug Bouma S, Tryggestad SS, Haukas E, Slordahl TS (2023). Comprehensive small RNA-sequencing of primary myeloma cells identifies miR-105-5p as a predictor of patient survival. Br J Cancer.

[24] Cox J, Mann M (2008). MaxQuant enables high peptide identification rates, individualized p.p.b.-range mass accuracies and proteome-wide protein quantification. Nat Biotechnol.

[25] Tyanova S, Albrechtsen R, Kronqvist P, Cox J, Mann M, Geiger T (2016). Proteomic maps of breast cancer subtypes. Nat Commun.

[26] Deeb SJ, D'Souza RC, Cox J, Schmidt-Supprian M, Mann M (2012). Super-SILAC allows classification of diffuse large B-cell lymphoma subtypes by their protein expression profiles. Mol Cell Proteomics.

[27] Tyanova S, Temu T, Cox J (2016). The MaxQuant computational platform for mass spectrometry-based shotgun proteomics. Nat Protoc.

[28] Geiszler DJ, Kong AT, Avtonomov DM, Yu F, Leprevost FDV, Nesvizhskii AI (2021). PTM-shepherd: analysis and summarization of post-translational and chemical modifications from open search results. Mol Cell Proteomics.

[29] Cox J, Hein MY, Luber CA, Paron I, Nagaraj N, Mann M (2014). Accurate proteome-wide label-free quantification by delayed normalization and maximal peptide ratio extraction, termed MaxLFQ. Mol Cell Proteomics.

[30] Tusher VG, Tibshirani R, Chu G (2001). Significance analysis of microarrays applied to the ionizing radiation response. Proc Natl Acad Sci USA.

[31] Misund K, Hofste Op Bruinink D, Coward E, Hoogenboezem RM, Rustad EH, Sanders MA (2022). Clonal evolution after treatment pressure in multiple myeloma: heterogenous genomic aberrations and transcriptomic convergence. Leukemia.

[32] Misund K, Keane N, Stein CK, Asmann YW, Day G, Welsh S (2020). MYC dysregulation in the progression of multiple myeloma. Leukemia.

[33] Chng WJ, Huang GF, Chung TH, Ng SB, Gonzalez-Paz N, Troska-Price T (2011). Clinical and biological implications of MYC activation: a common difference between MGUS and newly diagnosed multiple myeloma. Leukemia.

[34] Bustoros M, Sklavenitis-Pistofidis R, Park J, Redd R, Zhitomirsky B, Dunford AJ (2020). Genomic profiling of smoldering multiple myeloma identifies patients at a high risk of disease progression. J Clin Oncol.

[35] Chesi M, Robbiani DF, Sebag M, Chng WJ, Affer M, Tiedemann R (2008). AID-dependent activation of a MYC transgene induces multiple myeloma in a conditional mouse model of post-germinal center malignancies. Cancer Cell.

[36] Shaffer AL, Emre NC, Lamy L, Ngo VN, Wright G, Xiao W (2008). IRF4 addiction in multiple myeloma. Nature.

[37] Kronke J, Udeshi ND, Narla A, Grauman P, Hurst SN, McConkey M (2014). Lenalidomide causes selective degradation of IKZF1 and IKZF3 in multiple myeloma cells. Science.

[38] Lu G, Middleton RE, Sun H, Naniong M, Ott CJ, Mitsiades CS (2014). The myeloma drug lenalidomide promotes the cereblon-dependent destruction of Ikaros proteins. Science.

[39] Katz LH, Li Y, Chen JS, Munoz NM, Majumdar A, Chen J (2013). Targeting TGF-beta signaling in cancer. Expert Opin Ther Targets.

[40] Ito S (2020). Proteasome inhibitors for the treatment of multiple myeloma. Cancers.

[41] Nakao A, Yoshihama M, Kenmochi N (2004). RPG: the ribosomal protein gene database. Nucl Acids Res.

[42] Daugeron MC, Lenstra TL, Frizzarin M, El Yacoubi B, Liu X, Baudin-Baillieu A (2011). Gcn4 misregulation reveals a direct role for the evolutionary conserved EKC/KEOPS in the t6A modification of tRNAs. Nucl Acids Res.

[43] Rojas-Benitez D, Eggers C, Glavic A (2017). Modulation of the proteostasis machinery to overcome stress caused by diminished levels of t6A-Modified tRNAs in drosophila. Biomolecules.

[44] Zykova T, Zhu F, Wang L, Li H, Lim DY, Yao K (2018). Targeting PRPK function blocks colon cancer metastasis. Mol Cancer Ther.

[45] Hideshima T, Cottini F, Nozawa Y, Seo HS, Ohguchi H, Samur MK (2017). p53-related protein kinase confers poor prognosis and represents a novel therapeutic target in multiple myeloma. Blood.

[46] Hu M, Fu X, Si Z, Li C, Sun J, Du X (2019). Identification of differently expressed genes associated with prognosis and growth in colon adenocarcinoma based on integrated bioinformatics analysis. Front Genet.

[47] Liu Y, Zhou J, Li X, Zhang X, Shi J, Wang X (2022). tRNA-m(1)A modification promotes T cell expansion via efficient MYC protein synthesis. Nat Immunol.

[48] Motorin Y, Quinternet M, Rhalloussi W, Marchand V (2021). Constitutive and variable 2'-O-methylation (Nm) in human ribosomal RNA. RNA Biol.

[49] Baldini L, Charpentier B, Labialle S (2021). Emerging data on the diversity of molecular mechanisms involving C/D snoRNAs. Noncoding RNA.

[50] Kelly EK, Czekay DP, Kothe U (2019). Base-pairing interactions between substrate RNA and H/ACA guide RNA modulate the kinetics of pseudouridylation, but not the affinity of substrate binding by H/ACA small nucleolar ribonucleoproteins. RNA.

[51] Licht K, Hartl M, Amman F, Anrather D, Janisiw MP, Jantsch MF (2019). Inosine induces context-dependent recoding and translational stalling. Nucl Acids Res.

[52] Zhang Z, Carmichael GG (2001). The fate of dsRNA in the nucleus: a p54(nrb)-containing complex mediates the nuclear retention of promiscuously A-to-I edited RNAs. Cell.

[53] Sun H, Li K, Liu C, Yi C (2023). Regulation and functions of non-m(6)A mRNA modifications. Nat Rev Mol Cell Biol.

[54] Sendinc E, Valle-Garcia D, Dhall A, Chen H, Henriques T, Navarrete-Perea J (2019). PCIF1 catalyzes m6Am mRNA methylation to regulate gene expression. Mol Cell.

[55] Winograd-Katz SE, Fassler R, Geiger B, Legate KR (2014). The integrin adhesome: from genes and proteins to human disease. Nat Rev Mol Cell Biol.

[56] Chen Z, Oh D, Dubey AK, Yao M, Yang B, Groves JT (2018). EGFR family and Src family kinase interactions: mechanics matters?. Curr Opin Cell Biol.

[57] Wolfenson H, Meacci G, Liu S, Stachowiak MR, Iskratsch T, Ghassemi S (2016). Tropomyosin controls sarcomere-like contractions for rigidity sensing and suppressing growth on soft matrices. Nat Cell Biol.

[58] Hosen N (2020). Integrins in multiple myeloma. Inflamm Regen.

[59] Chu H, Awasthi A, White GC, Chrzanowska-Wodnicka M, Malarkannan S (2008). Rap1b regulates B cell development, homing, and T cell-dependent humoral immunity. J Immunol.

[60] Duchniewicz M, Zemojtel T, Kolanczyk M, Grossmann S, Scheele JS, Zwartkruis FJ (2006). Rap1A-deficient T and B cells show impaired integrin-mediated cell adhesion. Mol Cell Biol.

[61] Berlin C, Berg EL, Briskin MJ, Andrew DP, Kilshaw PJ, Holzmann B (1993). Alpha 4 beta 7 integrin mediates lymphocyte binding to the mucosal vascular addressin MAdCAM-1. Cell.

[62] Katayama Y, Hidalgo A, Peired A, Frenette PS (2004). Integrin alpha4beta7 and its counterreceptor MAdCAM-1 contribute to hematopoietic progenitor recruitment into bone marrow following transplantation. Blood.

[63] Neri P, Ren L, Azab AK, Brentnall M, Gratton K, Klimowicz AC (2011). Integrin beta7-mediated regulation of multiple myeloma cell adhesion, migration, and invasion. Blood.

[64] Roy Choudhury S, Byrum SD, Alkam D, Ashby C, Zhan F, Tackett AJ (2023). Expression of integrin beta-7 is epigenetically enhanced in multiple myeloma subgroups with high-risk cytogenetics. Clin Epigenet.

[65] Hosen N, Matsunaga Y, Hasegawa K, Matsuno H, Nakamura Y, Makita M (2017). The activated conformation of integrin beta(7) is a novel multiple myeloma-specific target for CAR T cell therapy. Nat Med.

[66] Song S, Zhang J, Su Q, Zhang W, Jiang Y, Fan G (2021). Downregulation of ITGA6 confers to the invasion of multiple myeloma and promotes progression to plasma cell leukaemia. Br J Cancer.

[67] Lonial S, Lee HC, Badros A, Trudel S, Nooka AK, Chari A (2021). Longer term outcomes with single-agent belantamab mafodotin in patients with relapsed or refractory multiple myeloma: 13-month follow-up from the pivotal DREAMM-2 study. Cancer.

[68] Holstein SA (2023). Ciltacabtagene autoleucel for the treatment of multiple myeloma. Drugs Today.

[69] Hansen DK, Sidana S, Peres LC, Colin Leitzinger C, Shune L, Shrewsbury A (2023). Idecabtagene vicleucel for relapsed/refractory multiple myeloma: real-world experience from the myeloma CAR T consortium. J Clin Oncol.

[70] Johnson. TJPCoJ. Janssen marks first approval worldwide for TECVAYLI® (teclistamab) with EC authorisation of first-in-class bispecific antibody for the treatment of patients with multiple myeloma. [Press release]. 2022. https://www.jnj.com/janssen-marks-first-approval-worldwide-for-tecvayli-teclistamab-with-ec-authorisation-of-first-in-class-bispecific-antibody-for-the-treatment-of-patients-with-multiple-myeloma.

[71] Grosicki S, Bednarczyk M, Kociszewska K (2023). Elranatamab: a new promising BispAb in multiple myeloma treatment. Expert Rev Anticancer Ther.

[72] Patino-Escobar B, Ferguson ID, Wiita AP (2022). Unraveling the surface proteomic profile of multiple myeloma to reveal new immunotherapeutic targets and markers of drug resistance. Cell Stress.

[73] Nijhof IS, Groen RW, Lokhorst HM, van Kessel B, Bloem AC, van Velzen J (2015). Upregulation of CD38 expression on multiple myeloma cells by all-trans retinoic acid improves the efficacy of daratumumab. Leukemia.

[74] Gomez-Herranz M, Taylor J, Sloan RD (2023). IFITM proteins: understanding their diverse roles in viral infection, cancer, and immunity. J Biol Chem.

[75] Borset M, Elsaadi S, Vandsemb EN, Hess ES, Steiro IJ, Cocera Fernandez M (2022). Highly expressed genes in multiple myeloma cells—what can they tell us about the disease?. Eur J Haematol.

[76] Ely SA, Knowles DM (2002). Expression of CD56/neural cell adhesion molecule correlates with the presence of lytic bone lesions in multiple myeloma and distinguishes myeloma from monoclonal gammopathy of undetermined significance and lymphomas with plasmacytoid differentiation. Am J Pathol.

[77] Lewinsky H, Gunes EG, David K, Radomir L, Kramer MP, Pellegrino B (2021). CD84 is a regulator of the immunosuppressive microenvironment in multiple myeloma. JCI Insight.

[78] Savage B, Saldivar E, Ruggeri ZM (1996). Initiation of platelet adhesion by arrest onto fibrinogen or translocation on von Willebrand factor. Cell.

[79] Shi Q, Ji T, Tang X, Guo W (2023). The role of tumor-platelet interplay and micro tumor thrombi during hematogenous tumor metastasis. Cell Oncol (Dordr).

[80] Jiang S, Zhou F, Zhang Y, Zhou W, Zhu L, Zhang M (2020). Identification of tumorigenicity-associated genes in osteosarcoma cell lines based on bioinformatic analysis and experimental validation. J Cancer.

[81] Martins Castanheira N, Spanhofer AK, Wiener S, Bobe S, Schillers H (2022). Uptake of platelets by cancer cells and recycling of the platelet protein CD42a. J Thromb Haemost.

[82] Rodriguez-Martinez A, Simon-Saez I, Perales S, Garrido-Navas C, Russo A, de Miguel-Perez D (2022). Exchange of cellular components between platelets and tumor cells: impact on tumor cells behavior. Theranostics.

[83] Tzeng HT, Wang YC (2016). Rab-mediated vesicle trafficking in cancer. J Biomed Sci.

[84] Han MZ, Huang B, Chen AJ, Zhang X, Xu R, Wang J (2017). High expression of RAB43 predicts poor prognosis and is associated with epithelial-mesenchymal transition in gliomas. Oncol Rep.

[85] Chen SN, Mai ZY, Mai JN, Liang W, Dong ZX, Ju FE (2023). E2F2 modulates cell adhesion through the transcriptional regulation of PECAM1 in multiple myeloma. Br J Haematol.

[86] Irie-Sasaki J, Sasaki T, Penninger JM (2003). CD45 regulated signaling pathways. Curr Top Med Chem.

[87] Bataille R, Robillard N, Pellat-Deceunynck C, Amiot M (2003). A cellular model for myeloma cell growth and maturation based on an intraclonal CD45 hierarchy. Immunol Rev.

[88] Abdollahi P, Kohn M, Borset M (2021). Protein tyrosine phosphatases in multiple myeloma. Cancer Lett.

[89] Baughn LB, Jessen E, Sharma N, Tang H, Smadbeck JB, Long MD (2023). Mass cytometry reveals unique phenotypic patterns associated with subclonal diversity and outcomes in multiple myeloma. Blood Cancer J.

[90] Descamps G, Pellat-Deceunynck C, Szpak Y, Bataille R, Robillard N, Amiot M (2004). The magnitude of Akt/phosphatidylinositol 3'-kinase proliferating signaling is related to CD45 expression in human myeloma cells. J Immunol.

[91] Chen WC, Hsu HP, Li CY, Yang YJ, Hung YH, Cho CY (2016). Cancer stem cell marker CD90 inhibits ovarian cancer formation via beta3 integrin. Int J Oncol.

[92] Salama Y, Heida AH, Yokoyama K, Takahashi S, Hattori K, Heissig B (2020). The EGFL7-ITGB3-KLF2 axis enhances survival of multiple myeloma in preclinical models. Blood Adv.

[93] Daverkausen-Fischer L, Draga M, Prols F (2022). Regulation of translation, translocation, and degradation of proteins at the membrane of the endoplasmic reticulum. Int J Mol Sci.

[94] Imai K, Slupphaug G, Lee WI, Revy P, Nonoyama S, Catalan N (2003). Human uracil-DNA glycosylase deficiency associated with profoundly impaired immunoglobulin class-switch recombination. Nat Immunol.

[95] Luo S, Qiao R, Zhang X (2022). DNA damage response and repair in adaptive immunity. Front Cell Dev Biol.

[96] Gourzones C, Bret C, Moreaux J (2019). Treatment may be harmful: mechanisms/prediction/prevention of drug-induced DNA damage and repair in multiple myeloma. Front Genet.

[97] Sousa MM, Zub KA, Aas PA, Hanssen-Bauer A, Demirovic A, Sarno A (2013). An inverse switch in DNA base excision and strand break repair contributes to melphalan resistance in multiple myeloma cells. PLoS ONE.

[98] Chen Q, Van der Sluis PC, Boulware D, Hazlehurst LA, Dalton WS (2005). The FA/BRCA pathway is involved in melphalan-induced DNA interstrand cross-link repair and accounts for melphalan resistance in multiple myeloma cells. Blood.

[99] Wood R LM. Human DNA repair genes. Update of the table cited in Wood RD, Mitchell M, & Lindahl T Mutation Research, 2005, in Science, 2001, in the reference book DNA Repair and Mutagenesis, 2nd edition, 2006, and in Nature Reviews Cancer, 2011 2020. https://www.mdanderson.org/documents/Labs/Wood-Laboratory/human-dna-repair-genes.html#chr.

[100] Parsons JL, Dianov GL (2013). Co-ordination of base excision repair and genome stability. DNA Repair (Amst).

[101] Vitor AC, Huertas P, Legube G, de Almeida SF (2020). Studying DNA double-strand break repair: an ever-growing toolbox. Front Mol Biosci.

[102] Botrugno OA, Bianchessi S, Zambroni D, Frenquelli M, Belloni D, Bongiovanni L (2020). ATR addiction in multiple myeloma: synthetic lethal approaches exploiting established therapies. Haematologica.

[103] Mjelle R, Hegre SA, Aas PA, Slupphaug G, Drablos F, Saetrom P (2015). Cell cycle regulation of human DNA repair and chromatin remodeling genes. DNA Repair.

[104] Zhan F, Huang Y, Colla S, Stewart JP, Hanamura I, Gupta S (2006). The molecular classification of multiple myeloma. Blood.

[105] Cottini F, Hideshima T, Suzuki R, Tai YT, Bianchini G, Richardson PG (2015). Synthetic lethal approaches exploiting DNA damage in aggressive myeloma. Cancer Discov.

[106] Wang LW, Jiang S, Yuan YH, Duan J, Mao ND, Hui Z (2022). Recent advances in synergistic antitumor effects exploited from the inhibition of ataxia telangiectasia and RAD3-related protein kinase (ATR). Molecules.

[107] Hunia J, Gawalski K, Szredzka A, Suskiewicz MJ, Nowis D (2022). The potential of PARP inhibitors in targeted cancer therapy and immunotherapy. Front Mol Biosci.

[108] Maura F, Degasperi A, Nadeu F, Leongamornlert D, Davies H, Moore L (2019). A practical guide for mutational signature analysis in hematological malignancies. Nat Commun.

[109] Zub KA, Sousa MM, Sarno A, Sharma A, Demirovic A, Rao S (2015). Modulation of cell metabolic pathways and oxidative stress signaling contribute to acquired melphalan resistance in multiple myeloma cells. PLoS ONE.

[110] Caracciolo D, Scionti F, Juli G, Altomare E, Golino G, Todoerti K (2021). Exploiting MYC-induced PARPness to target genomic instability in multiple myeloma. Haematologica.

[111] Shen HY, Tang HL, Zheng YH, Feng J, Dong BX, Chen XQ (2022). The PARP1 inhibitor niraparib represses DNA damage repair and synergizes with temozolomide for antimyeloma effects. J Oncol.

[112] Soumerai JD, Zelenetz AD, Moskowitz CH, Palomba ML, Hamlin PA, Noy A (2017). The PARP inhibitor veliparib can be safely added to bendamustine and rituximab and has preliminary evidence of activity in B-Cell lymphoma. Clin Cancer Res.

[113] Wu Z, Huang Y, Yuan W, Wu X, Shi H, Lu M (2022). Expression, tumor immune infiltration, and prognostic impact of HMGs in gastric cancer. Front Oncol.

[114] Pellarin I, Arnoldo L, Costantini S, Pegoraro S, Ros G, Penzo C (2016). The architectural chromatin factor high mobility group A1 enhances DNA ligase IV activity influencing DNA repair. PLoS ONE.

[115] Pentimalli F, Palmieri D, Pacelli R, Garbi C, Cesari R, Martin E (2008). HMGA1 protein is a novel target of the ATM kinase. Eur J Cancer.

[116] Palmieri D, Valentino T, D'Angelo D, De Martino I, Postiglione I, Pacelli R (2011). HMGA proteins promote ATM expression and enhance cancer cell resistance to genotoxic agents. Oncogene.

[117] Wen B, Wei YT, Zhao K (2021). The role of high mobility group protein B3 (HMGB3) in tumor proliferation and drug resistance. Mol Cell Biochem.

[118] Guggenheim ER, Xu D, Zhang CX, Chang PV, Lippard SJ (2009). Photoaffinity isolation and identification of proteins in cancer cell extracts that bind to platinum-modified DNA. ChemBioChem.

[119] Mukherjee A, Huynh V, Gaines K, Reh WA, Vasquez KM (2019). Targeting the high-mobility group box 3 protein sensitizes chemoresistant ovarian cancer cells to cisplatin. Cancer Res.

[120] Masaoka A, Gassman NR, Kedar PS, Prasad R, Hou EW, Horton JK (2012). HMGN1 protein regulates poly(ADP-ribose) polymerase-1 (PARP-1) self-PARylation in mouse fibroblasts. J Biol Chem.

[121] Kim YC, Gerlitz G, Furusawa T, Catez F, Nussenzweig A, Oh KS (2009). Activation of ATM depends on chromatin interactions occurring before induction of DNA damage. Nat Cell Biol.

[122] Buhr F, Jha S, Thommen M, Mittelstaet J, Kutz F, Schwalbe H (2016). Synonymous codons direct cotranslational folding toward different protein conformations. Mol Cell.

[123] Erdal E, Haider S, Rehwinkel J, Harris AL, McHugh PJ (2017). A prosurvival DNA damage-induced cytoplasmic interferon response is mediated by end resection factors and is limited by Trex1. Genes Dev.

[124] Song P, Yang W, Lou KF, Dong H, Zhang H, Wang B (2022). UNC13D inhibits STING signaling by attenuating its oligomerization on the endoplasmic reticulum. EMBO Rep.

[125] Blade J, Esteve J (2000). Viewpoint on the impact of interferon in the treatment of multiple myeloma: benefit for a small proportion of patients?. Med Oncol.

[126] Mushinski JF, Nguyen P, Stevens LM, Khanna C, Lee S, Chung EJ (2009). Inhibition of tumor cell motility by the interferon-inducible GTPase MxA. J Biol Chem.

[127] Choi HJ, Lui A, Ogony J, Jan R, Sims PJ, Lewis-Wambi J (2015). Targeting interferon response genes sensitizes aromatase inhibitor resistant breast cancer cells to estrogen-induced cell death. Breast Cancer Res.

[128] Callari M, Musella V, Di Buduo E, Sensi M, Miodini P, Dugo M (2014). Subtype-dependent prognostic relevance of an interferon-induced pathway metagene in node-negative breast cancer. Mol Oncol.

[129] Heink S, Ludwig D, Kloetzel PM, Kruger E (2005). IFN-gamma-induced immune adaptation of the proteasome system is an accelerated and transient response. Proc Natl Acad Sci USA.

[130] Johnston-Carey HK, Pomatto LC, Davies KJ (2015). The Immunoproteasome in oxidative stress, aging, and disease. Crit Rev Biochem Mol Biol.

[131] Pickering AM, Davies KJ (2012). Differential roles of proteasome and immunoproteasome regulators Pa28alphabeta, Pa28gamma and Pa200 in the degradation of oxidized proteins. Arch Biochem Biophys.

[132] Atanackovic D, Hildebrandt Y, Templin J, Cao Y, Keller C, Panse J (2012). Role of interleukin 16 in multiple myeloma. J Natl Cancer Inst.

[133] Templin J, Atanackovic D, Hasche D, Radhakrishnan SV, Luetkens T (2017). Oscillating expression of interleukin-16 in multiple myeloma is associated with proliferation, clonogenic growth, and PI3K/NFKB/MAPK activation. Oncotarget.

[134] Chang Y, Hsiao YM, Hu CC, Chang CH, Li CY, Ueng SWN (2020). Synovial fluid interleukin-16 contributes to osteoclast activation and bone loss through the JNK/NFATc1 signaling cascade in patients with periprosthetic joint infection. Int J Mol Sci.

[135] Franke NE, Niewerth D, Assaraf YG, van Meerloo J, Vojtekova K, van Zantwijk CH (2012). Impaired bortezomib binding to mutant beta5 subunit of the proteasome is the underlying basis for bortezomib resistance in leukemia cells. Leukemia.

[136] Lichter DI, Danaee H, Pickard MD, Tayber O, Sintchak M, Shi H (2012). Sequence analysis of beta-subunit genes of the 20S proteasome in patients with relapsed multiple myeloma treated with bortezomib or dexamethasone. Blood.

[137] Leung-Hagesteijn C, Erdmann N, Cheung G, Keats JJ, Stewart AK, Reece DE (2013). Xbp1s-negative tumor B cells and pre-plasmablasts mediate therapeutic proteasome inhibitor resistance in multiple myeloma. Cancer Cell.

[138] Soriano GP, Besse L, Li N, Kraus M, Besse A, Meeuwenoord N (2016). Proteasome inhibitor-adapted myeloma cells are largely independent from proteasome activity and show complex proteomic changes, in particular in redox and energy metabolism. Leukemia.

[139] Tsherniak A, Vazquez F, Montgomery PG, Weir BA, Kryukov G, Cowley GS (2017). Defining a cancer dependency map. Cell.

[140] Ferguson ID, Lin YT, Lam C, Shao H, Tharp KM, Hale M (2022). Allosteric HSP70 inhibitors perturb mitochondrial proteostasis and overcome proteasome inhibitor resistance in multiple myeloma. Cell Chem Biol.

[141] Vizcaino JA, Csordas A, Del-Toro N, Dianes JA, Griss J, Lavidas I (2016). 2016 update of the PRIDE database and its related tools. Nucl Acids Res.

[142] Drew K, Muller CL, Bonneau R, Marcotte EM (2017). Identifying direct contacts between protein complex subunits from their conditional dependence in proteomics datasets. PLoS Comput Biol.

