# Supplementary material for: Progression of monoclonal gammopathy of undetermined significance to multiple myeloma is associated with enhanced translational quality control and overall loss of surface antigens
Source: J Transl Med. 2024 Jun 7;22:548. doi: 10.1186/s12967-024-05345-x (PMC11162064; doi:10.1186/s12967-024-05345-x)
Supplement: Supplementary file 1 — Supplementary Material1 (PDF 1322 KB) [file 12967_2024_5345_MOESM1_ESM.pdf]

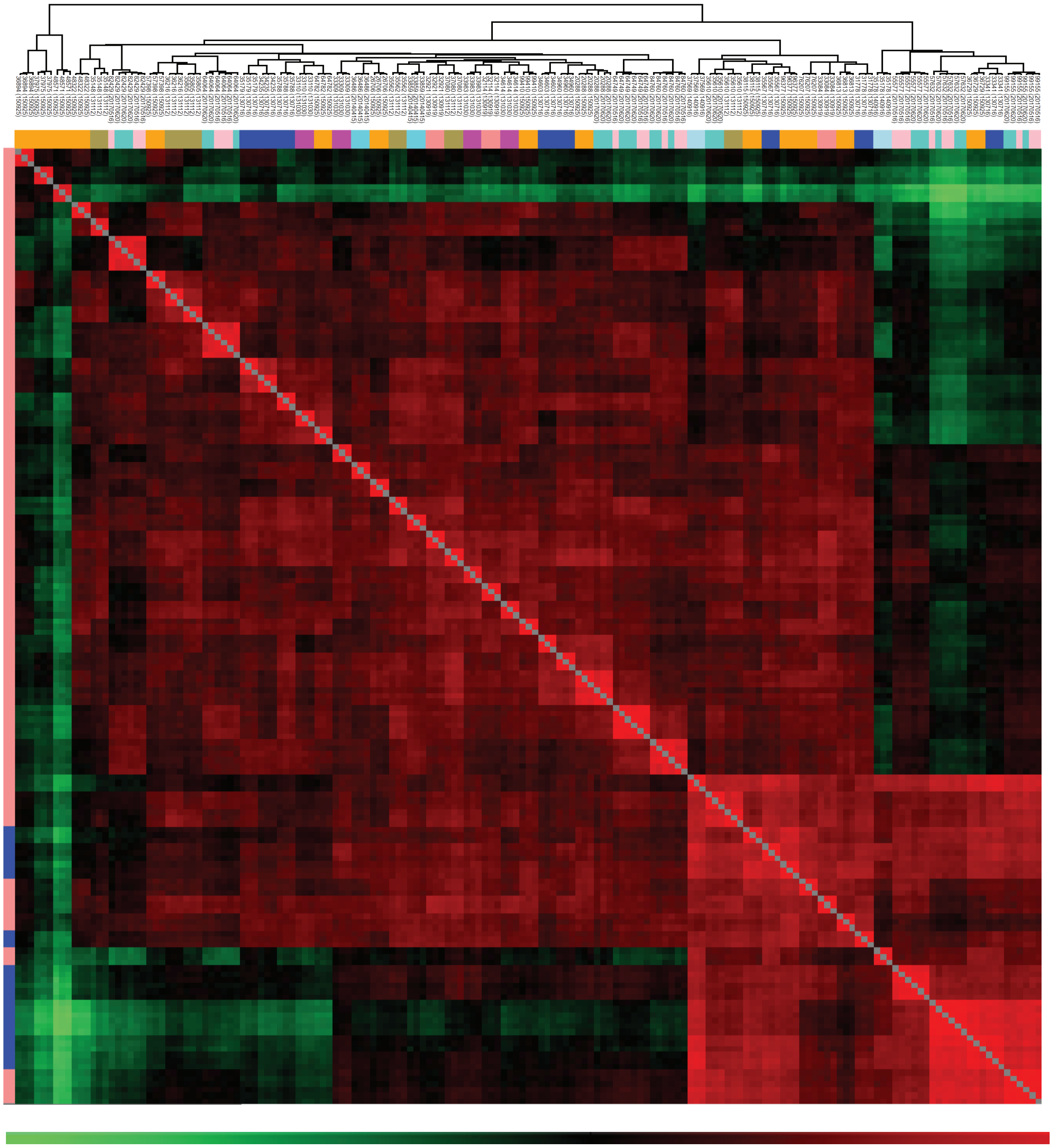

Supplementary Figure 1. Overall clustering with technical replicates and batch-information shown coloured on columns and patient groups (MM as peach and MGUS as blue) on rows.
