# Supplementary material for: Progression of monoclonal gammopathy of undetermined significance to multiple myeloma is associated with enhanced translational quality control and overall loss of surface antigens
Source: J Transl Med. 2024 Jun 7;22:548. doi: 10.1186/s12967-024-05345-x (PMC11162064; doi:10.1186/s12967-024-05345-x)
Supplement: Supplementary file 2 — Supplementary Material2 (PDF 328 KB) [file 12967_2024_5345_MOESM2_ESM.pdf]

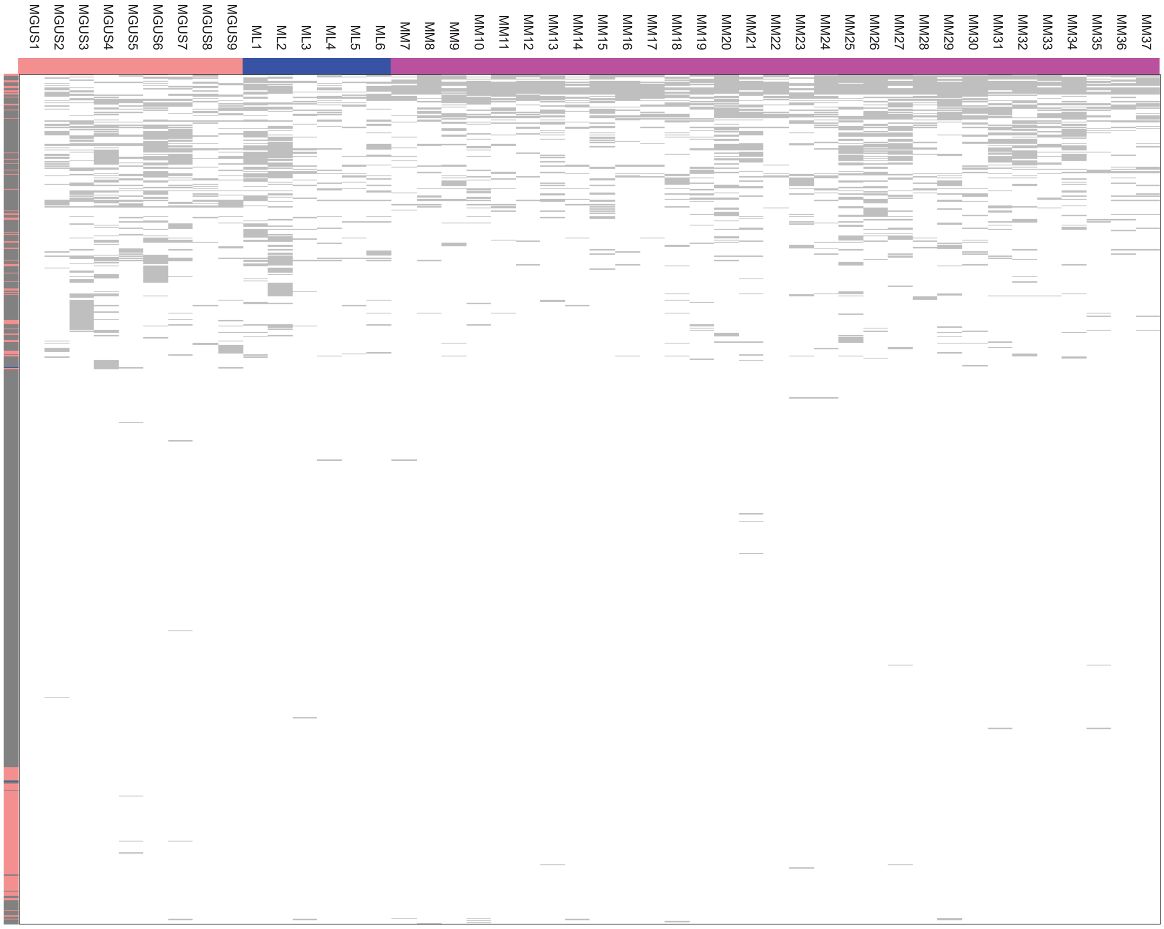

Supplementary Figure 2 . Out of about 150000 values, about 10000 were imputed at an average of 3.3 per row with median of 0 values imputed. Most of the imputed values do not seem to contribute much to the test of significance (missing values marked as grey bands) where protein-groups/rows which appear significant are marked as peach (MM vs. MGUS) and blue (MM vs. ML).
