# Supplementary material for: Progression of monoclonal gammopathy of undetermined significance to multiple myeloma is associated with enhanced translational quality control and overall loss of surface antigens
Source: J Transl Med. 2024 Jun 7;22:548. doi: 10.1186/s12967-024-05345-x (PMC11162064; doi:10.1186/s12967-024-05345-x)
Supplement: Supplementary file 3 — Supplementary Material3 (PDF 343 KB) [file 12967_2024_5345_MOESM3_ESM.pdf]

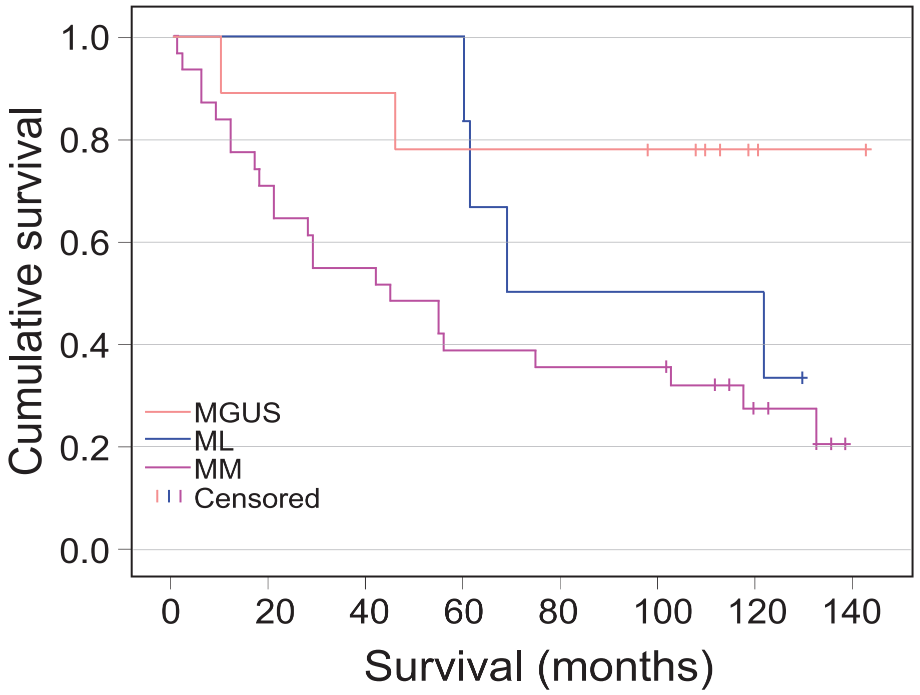

	Mean[a]				Median			
			95% confidence interval				95% confidence interval	
Category	Estimate	Std. error	Lower bound	Upper bound	Estimate	Std. error	Lower bound	Upper bound
MGUS	117.44	16.19	85.72	149.17				
ML	95.33	13.16	69.54	121.13	69.00	37.36	0.00	142.22
MM	64.06	9.57	45.31	82.82	45.00	12.52	20.46	69.54
Overall	78.91	8.16	62.93	94.90	61.00	26.67	8.74	113.26

[a]Estimation is limited to the largest survival time if it is censored

Supplementary Figure 3. Kaplan-Meier curves for overall survival in the MGUS, ML and MM groups, with statistics indicated below. The crossing over of MGUS and ML at 60 months is caused by the early death of two patients in the MGUS cohort, apparently by non-MGUS related causes. One of these died of pneumonia-induced septic shock 10 months after diagosis. The other most likely died by natural causes 46 months after diagnosis, at the age of 88.
